# Circadian misalignment by environmental light/dark shifting causes circadian disruption in colon

**DOI:** 10.1371/journal.pone.0251604

**Published:** 2021-06-04

**Authors:** Laura Tran, Sarah B. Jochum, Maliha Shaikh, Sherry Wilber, Lijuan Zhang, Dana M. Hayden, Christopher B. Forsyth, Robin M. Voigt, Faraz Bishehsari, Ali Keshavarzian, Garth R. Swanson

**Affiliations:** 1 Rush Medical College, Rush Center for Integrated Microbiome and Chronobiology Research, Rush University Medical Center, Chicago, Illinois, United States of America; 2 Department of Surgery, Rush University Medical Center, Chicago, Illinois, United States of America; 3 Department of Medicine, Rush University Medical Center, Chicago, Illinois, United States of America; Karlsruhe Institute of Technology, GERMANY

## Abstract

**Background:**

Physiological circadian rhythms (CRs) are complex processes with 24-hour oscillations that regulate diverse biological functions. Chronic weekly light/dark (LD) shifting (CR disruption; CRD) in mice results in colonic hyperpermeability. However, the mechanisms behind this phenomenon are incompletely understood. One potential innovative *in vitro* method to study colonic CRs are colon organoids. The goals of this study were to utilize circadian clock gene Per2 luciferase reporter (Per2::Luc) mice to measure the effects of chronic LD shifting on colonic tissue circadian rhythmicity *ex vivo* and to determine if organoids made from shifted mice colons recapitulate the *in vivo* phenotype.

**Methods:**

Non-shifted (NS) and shifted (S) BL6 Per2::Luc mice were compared after a 22-week experiment. NS mice had a standard 12h light/12h dark LD cycle throughout. S mice alternated 12h LD patterns weekly, with light from 6am-6pm one week followed by shifting light to 6pm-6am the next week for 22 weeks. Mice were tested for intestinal permeability while colon tissue and organoids were examined for CRs of bioluminescence and proteins of barrier function and cell fate.

**Results:**

There was no absolute difference in NS vs. S 24h circadian period or phase. However, chronic LD shifting caused Per2::Luc S mice colon tissue to exhibit significantly greater variability in both the period and phase of Per2::Luc rhythms than NS mice colon tissue and organoids. Chronic LD shifting also resulted in increased colonic permeability of the Per2::Luc mice as well as decreased protein markers of intestinal permeability in colonic tissue and organoids from shifted Per2:Luc mice.

**Conclusions:**

Our studies support a model in which chronic central circadian disruption by LD shifting alters the circadian phenotype of the colon tissue and results in colon leakiness and loss of colonic barrier function. These CRD-related changes are stably expressed in colon stem cell derived organoids from CRD mice.

## Introduction

Physiological circadian rhythms are complex biological processes with 24-hour oscillations that regulate diverse bodily functions, including metabolism, regeneration, immunology, endocrinology, and behavior. These rhythms are entrained to environmental cues like light/dark (LD) cycles [1, 2] and are orchestrated by the central circadian clock as well as peripheral circadian machinery present in all human tissues [3–5]. The molecular circadian clock is comprised of a transcriptional/translational feedback loop of core clock genes, including *Clock*, *Bmal1*, *Cry1-2* and *Per1-3* [2, 5]. The focus of this study is on intestinal, specifically colonic epithelial, circadian rhythms [6, 7]. Multiple studies have shown that the intestinal tract demonstrates circadian rhythms and is susceptible to circadian disruption [6–8]. Disruption of normal circadian rhythm has been associated with many gastrointestinal (GI) pathologies including exacerbated irritable bowel disease (IBS), increased risk of colorectal cancer [9, 10], and GI complaints associated with jet lag [11]. *In vitro* studies have shown the circadian clock proteins regulate intestinal hyperpermeability in response to alcohol treatment and these studies are supported by data in alcohol fed mice [12]. In human studies, night shift workers with circadian disruption have decreased resiliency to colonic alcohol induced gut leakiness [13]. In rodents, chronic environmental circadian disruption of mice through LD shifting for 22 weeks also induces colonic hyperpermeability, or gut leakiness [14]. In this study, we will refer to circadian disruption as a reference to this chronic 22-week light/dark shifting model.

In each of these studies the greatest intestinal leakiness was found in the colon, also the site of the majority of the microbiome [6]. The colonic epithelial barrier protects the body from pro-inflammatory intestinal luminal contents, including bacterial endotoxins [15]. Maintenance of a healthy gut intestinal epithelial barrier requires a network of cell-cell junctions including tight junction proteins, adherens junctions, and desmosomes, collectively known as the apical junctional complex (AJC) [15, 16]. Any disruption of this barrier can result in gut leakiness, which has been associated with several pathologies involving the gastrointestinal tract, including alcoholic and nonalcoholic hepatic steatosis, inflammatory bowel disease, and IBS [14, 17–19]. Gut leakiness is also linked to diseases of other systems, including metabolic syndrome [20], diabetes [20–22], ankylosing spondylitis [23], cardiovascular disease [24], Parkinson’s disease [25], amyotrophic lateral sclerosis [26], and depression [27]. While circadian disruption and gut leakiness have been clearly linked, it is not completely understood how the specific components of the circadian clock regulate intestinal barrier homeostasis.

*In vitro* intestinal circadian rhythm studies using colonic tissue are challenging and can be limited by the quantity of tissue available per subject, survive a limited of time *in vitro*, and complex to analyze due to the heterogeneity of the various intestinal cell types. One exciting and innovative solution is to utilize colonic stem cell-derived organoids [28]. Colon organoids allow for growth of a long-term colonic epithelial organoid cultures from primary human or animal colon tissue without inducing a genetic transformation [29]. A single Lgr5-CBC stem cell or isolated crypts containing these cells can be used to grow three-dimensional colon epithelial organoids for *in vitro* culture that can last more than 1.5 years [30, 31]. Organoids are able to organize into crypt-like domains and differentiate, providing a more accurate model of cell-cell contacts and communication pathways that coordinate colonic epithelial circadian behaviors *in vivo*. This self-renewing source of colon epithelial cell types can be used for transfection of DNA or RNA [32], and analyzed by immunohistochemistry-immunofluorescence, gene expression microarray, and mass spectrometry [29]. Organoids may also be used for co-culturing, manipulation of cell fate, and experimental treatments. Per2 luciferase (Per2::Luc) reporter mice [33] have been used in the past to measure *ex vivo* cell culture circadian rhythms of fibroblasts [34], hepatocytes [35] and significantly in small intestinal epithelium-derived organoids [36]. The circadian rhythmicity of luciferase activity can then be assessed into three primary components: phase shift, period, and amplitude by frequent assessments over multiple 24-hour periods.

We hypothesized that colon organoids derived from environmentally circadian disrupted mice through chronic LD shifting would exhibit the same disrupted circadian rhythm as colon tissue from the same mice. To our knowledge, it has not yet been determined if: 1) colon epithelial organoids derived from Per2:Luc mice also demonstrate a circadian rhythm, and 2) whether colonic organoids maintain the phenotype of the host *ex vivo* (after 3 weeks in organoid culture) in a chronic environmental model of disrupted circadian rhythm similar to tissue.

The aims of this study were to utilize Per2::Luc reporter mice to examine the effects of chronic LD circadian disruption (22 weeks) on colonic tissue circadian rhythmicity and to determine if colonic epithelial organoids derived from circadian disrupted Per2:Luc mice colon tissue maintain the disrupted circadian phenotype of the host. Additionally, we used the Per2::Luc mouse organoid model to investigate if the loss of colonic epithelial AJC markers (ZO-1, E-cadherin) seen with gut leakiness due to LD shifting was maintained in organoids from these mice.

## Methods

### Mice LD phase shifting model

All animal studies in these experiments used a Rush University Medical Center (RUMC) IACUC approved protocol. At 21 days old, male C57BL/6J mice (hereafter BL6 mice) and homozygous Per2::Luc reporter BL6 mice [33] from Jackson Labs (Bar Harbor, ME) were bred at RUMC, weaned and group housed with 2–4 mice per cage. 6-8-week-old mice were then used for each experiment and placed in light-tight chambers as previously described [14]. Mice were all maintained on a 12h light/12h dark schedule. There were two groups per mouse type: non-shifted (NS) and shifted (S). The NS mice had a standard 12h LD cycle throughout the 22-week experiment. The shifted mice followed a pattern of adhering to a 6am-6pm light and 6pm-6am dark schedule during odd numbered weeks and were ‘shifted’ to the opposite LD schedule (6am-6pm dark, 6am-6pm light) on even numbered weeks for the 22 weeks of the experiment (**S1 Fig**). Both NS and S mice had free access to water and chow *ad libitum* throughout the study. All animal studies in these experiments used a Rush University Medical Center (RUMC) IACUC approved protocol. End of study euthanasia was performed at ZT0 using carbon dioxide (primary method) and cervical dislocation (secondary method).

For central circadian rhythm measurements of activity, both BL6 and Per2::Luc mice were provided food *ad libitum* and placed in singly housed cages with free rotating wheels at age 6–8 weeks [37]. To allow for acclimation to the wheel, mice were kept at the 12h LD cycle to which they were previously entrained for two weeks. Following two weeks of 12h LD, mice were kept in dark:dark (DD) ‘free-running’ conditions for two weeks. Under these conditions, mice had minimal disturbances, with brief randomly timed visual inspection with red light only. DD actigram data was analyzed following the completion of the two weeks using CLOCKLAB software (Actimetrics, Wilmette, IL).

### Per2-luciferase bioluminescence

The advantage of utilizing the Per2::Luc mice is that the circadian oscillations of luciferase-tagged Per2 in colonic tissue or colon epithelial cells can be captured using colon tissue or colon organoids in covered 35mm dishes with the eight-dish wheel Kronos Dio apparatus (AB-2500, Atto Co., Tokyo, Japan). The device quantified the bioluminescence expression levels of the Per2-luciferase protein in luciferin containing media measured for one minute of exposure every 10 minutes, with a controlled nominal temperature of 37°C/5%CO_2_.

### Human night shift worker model

IRB approval was obtained from the RUMC IRB. All subjects completed and signed RUMC IRB-approved informed consent forms. Night shift nurses and day shift nurses were asked to have flexible sigmoidoscopies at 8 am. Biopsies of normal appearing colon mucosa were performed.

Participants were recruited through a combination of Rush University Medical Center GI clinic, advertisements, posted flyers, or self-referral. Day Shift Workers (**DSW**) were healthy subjects (primarily nurses) who and had been on a stable work schedule of 7 am– 7pm for at least 3 shifts a week for greater than 3 months. Night shift workers were healthy subjects on a stable work schedule of 7 pm– 7 am of at least 3 shifts a week for greater than 3 months. All subjects completed blood tests and questionnaires at their initial visit and were excluded if they met any of the following criteria: 1) Inability to sign an informed consent; 2) Any history or blood tests consistent with liver disease (LFTs > 1.5 normal), renal impairment (Creatinine > 1.5), cardiovascular disease, diabetes (Hgb-A1c>8%), or thrombocytopenia (<80k); 3) Major depression (score ≥ 15 or any endorsement of suicidal intent on the Beck Depression Inventory) [38]; 4) Sleep apnea (score high risk ≥ 2 or more categories on the Berlin Questionnaire) [39] or 5) Restless leg syndrome IRLSSG consensus criteria for Restless Leg Syndrome.

### Intestinal permeability

*In vivo* intestinal permeability was determined at the end of 22 weeks as we have previously described [14, 40]. The test was performed at Zeitgeber time (ZT) 0 (lights on), after mice had been fasted for eight hours. A 200 μL solution containing lactulose (3.2 mg), sucrose (0.45 mg), sucralose (0.45 mg) and mannitol (0.9 mg) was orally gavaged, after which 2.0 mL of lactated ringers was administered subcutaneously to promote urine output. Mice were placed in metabolic cages for five hours, after which urine was collected and the total volume recorded. Intestinal permeability was calculated by using gas chromatography to measure urinary sugar concentrations. Permeability is expressed as percent urinary excretion of the oral dose of each sugar [40].

### Mouse organoid preparation and culture

The mouse intestinal epithelial organoid cultures from the colon were prepared using a method described by Sato et al. [30, 31, 41] as we have also published [42]. Briefly, the colon was washed with ice cold PBS without Ca^++^ and Mg^++^. The samples were cut longitudinally and gently scraped with a glass cover slip. The samples were washed with cold PBS twice and cut into small pieces, then transferred into a 50 mL conical tube. A further five washes were performed with PBS and then the samples were transferred into a separate 50 mL conical with 2.5 mM EDTA chelating buffer and placed on a shaker at 4°C for one hour. After the removal of the supernatant, the tissue pieces were washed in PBS and the first fraction was discarded. Three mL of PBS was added to the tubes and pipetted gently multiple times to loosen the crypts. Next, the crypts were filtered through a 70 μm cell strainer and saved in a fresh conical tube. This washing and filtering was repeated 3 times with each fraction added to the fresh conical tube. 10% FBS was added to the crypt suspension and the samples centrifuged for 5 min at 300g. The supernatant was discarded and the cell pellet resuspended in 15 mL of organoid media minus growth factors. As before, the cell suspension was centrifuged at 150g for 3 min and the supernatant was discarded. This organoid media washing and centrifugation step was repeated twice to get a clean crypt suspension. After the final centrifugation the crypts were counted and resuspended in cold Matrigel (60 μL per well) and plated on a warm 24 well culture plate. The plate was then incubated at 37°C for 30 min for the Matrigel to polymerize and then 500 μL of organoid medium with the growth factors was added to each well. The organoids were then incubated at 37°C/5% CO_2_ and allowed to grow for 21 days (time required for colonic organoids to grow).

### Immunofluorescent staining

The organoid immunofluorescent staining was performed on 8 well chamber slides (Nunc, Rochester, NY). Immunofluorescent staining occurred following at least 21 days (3 weeks) following colon isolation and organoid generation, to allow optimal colonic organoid growth. The medium was removed and the organoids in Matrigel were washed three times with cold PBS. Corning Cell Recovery Solution (500 μL) was added to the well and the Matrigel suspension transferred to a 15 mL conical tube. An additional 500 μL of Cell Recovery Solution was added to wash the well. The samples were kept on ice for 1 hour and tube inverted regularly to completely dissolve the Matrigel. The cells were then centrifuged at 300g for 5 min. Upon removal of the supernatant the cells were washed twice further with ice cold PBS. The organoids were fixed with warm 4% paraformaldehyde (PFA) on chamber slides for one hour at room temperature (RT) and then permeabilized with 1% Triton-X100. The samples were blocked with a solution of 3% goat serum/1% bovine serum albumin (BSA)/0.2% Triton X-100 for 1 hour. Primary staining for the organoids was done overnight at RT in a humidified chamber. The next day, organoids were washed and incubated for two hours with the appropriate secondary antibodies conjugated to Alexa Fluor 488. After washing, they were further stained for DAPI and mounted using Flouromount aqueous mounting medium (Sigma Aldrich). A Zeiss LSM 700 confocal microscope (Zeiss, Oberkochen, Germany) was used to image the organoids. Specific antibodies for staining are detailed in **S1 Table**. All organoid staining data is from n = 4 mice with images from at least 10 stained organoids or tissue section areas used to determine relative expression of each marker (using ImageJ) and to pick the representative images. All staining was evaluated by two blinded independent observers.

### Western blot analysis

For Western blot, either total proximal colon tissue or organoid protein was determined (Bio-Rad, Hercules, CA). Tissue samples were collected and immediately frozen in liquid nitrogen and stored at −80°C until use. Organoid protein was collected at least 21 days (3 weeks) following colon isolation and organoid generation to allow optimal colonic organoid growth. Laemmli sample buffer with 2-ME (Bio-Rad) was used to prepare the samples. Thirty micrograms of protein per lane were loaded into a 4%/10% stacking acrylamide Tris gel and electrophoresed at 100 V for two hours [43]. Membrane transfer, blocking, and primary and secondary antibody incubation were performed as previously described [44]. Chemiluminescent substrate (ECL, GE Healthcare) was then applied to the membrane for protein visualization with autoradiography film (HyBlot CL, Denville Scientific, Metuchen, NJ). Optical density was determined via densitometric analysis using Image J Software (NIH, Bethesda, MD). Specific antibodies are detailed in **S2 Table** and original uncropped and unadjusted images of blots and gel results are included in the Supporting Information section.

### Statistical analysis

Analysis of variance (ANOVA) was used to detect effects of shifting in permeability, apical junction complexes (AJC) and cell fate markers. Significance levels were set at p < 0.05 in all analyses. Statistics were performed using GraphPad, SPSS v 26 (IBM), and circular data was measured by Oriana v 4. Biodare 2 was used to analyze luciferase time series data and periodogram was performed to measure period, acrophase and amplitude by Fast Fourier Transformation Nonlinear Least Squares algorithm (FFT-NLLS) [45]. FFT-NLLS is a variation of the cosinor method and is reported to be less sensitive to missing or noisy data. The general equation for the cosinor fit is as follows [46, 47]:

Y(t)=M+Acos(2πt/τ+ϕ)

where M is the MESOR (Midline Statistic of Rhythm, a rhythm adjusted mean), A is the amplitude (a measure of half the extent of the variation within the cycle), *ϕ* is the acrophase (a measure of the time of overall highest value), and τ is the period.

Circular variance is calculated as follows: [V = 1-r]. Where r = length of the mean vector, and V = Circular variance. Circular variance has been found to an important marker in genetic models of circadian disruption [48]. All circular statistics were calculated using Oriana, v4.

## Results

### Central circadian rhythm

To further establish that Per2::Luc mice exhibit normal circadian function, we sought to measure the circadian phenotype of the Per2::Luc mice compared to the WT BL6 mice (**S2 Fig**). Activity was recorded on running wheels for 14 days in LD followed by 14 days in dark:dark (DD) for Per2::Luc and BL6 mice. The LD environment involves 12h light exposure from 6:00 am to 6:00 pm. The DD environment is constant darkness; therefore, mice express an endogenous circadian rhythm (free running) and period [37]. Based on wheel data in LD and DD environments, we found that Per2::Luc mice central circadian period (24.10±0.16 hours) differed slightly from WT BL6 mice at baseline (23.81±0.067 hours, p = 0.013) however the Per2::Luc mice were found to exhibit a clear and similarly shaped stable circadian rhythm curve to WT BL6 mice (**S2 Fig**). These data support that the Per2::Luc mice exhibit a normal circadian rhythm.

### Circadian related proteins functional binding to Per2 protein is not disrupted in Per2:Luc mice

**Fig 1:**
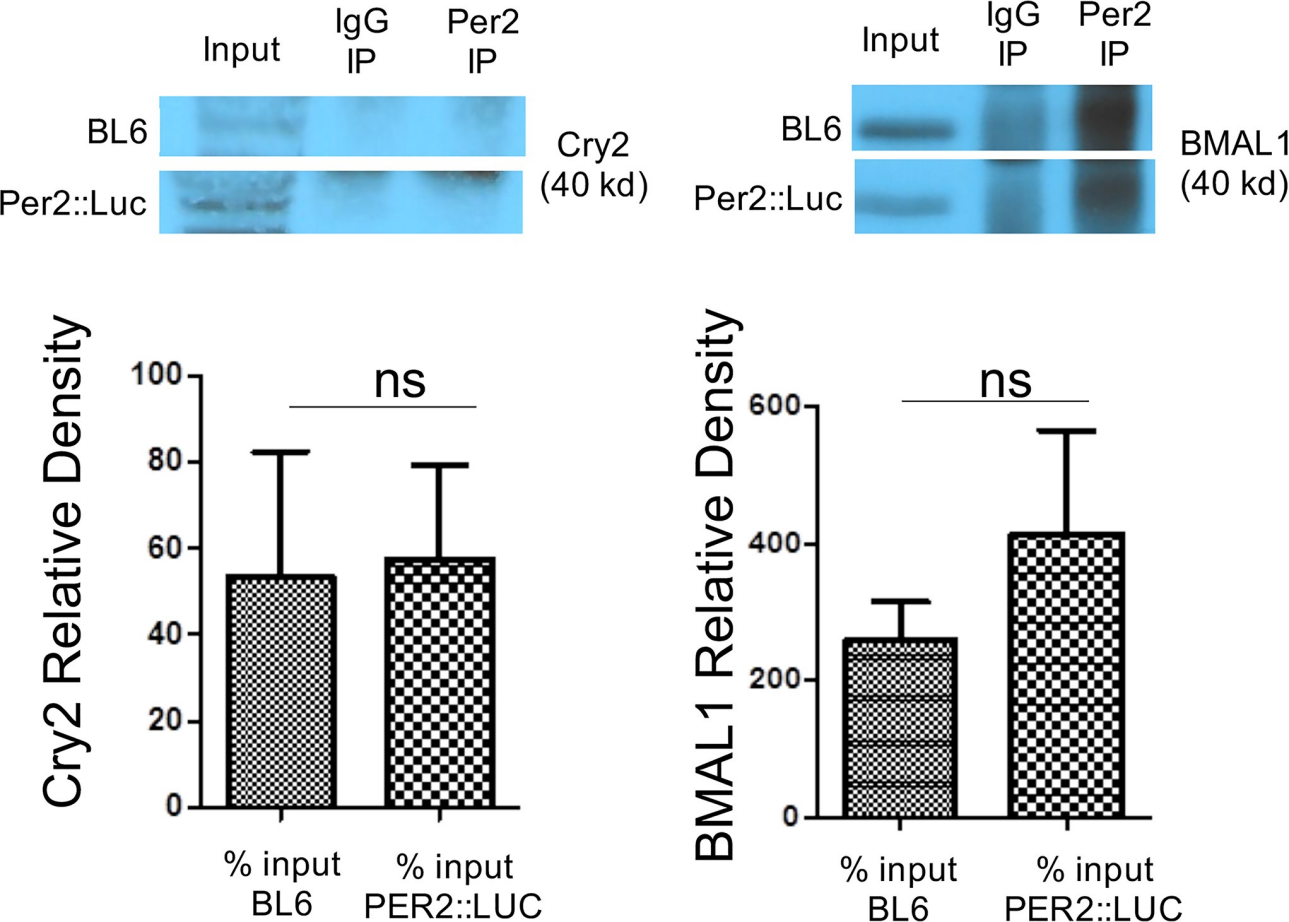
Colon tissue Per2::Luc mice Per2 protein displays typical interaction with Cry2 and Bmal1 circadian proteins. We compared interactions of Per2::Luc fusion protein vs. WTBL6 Per2 protein with well validated circadian protein binding partners for Per2, namely Cry2 and Bmal1 proteins using immunoprecipitation with Per2 Ab of colon tissue lysates. Data for Cry2 (left) and Bmal1 (right) show no significant difference in binding of either Cry2 or Bmal1 with the Per2::Luc protein vs. the normal BG proteins. The relative density of Cry2 and Bmal1 are represented for BL6 and Per2::Luc mice. There was no difference in colon tissue levels of Cry2 or Bmal1 levels at a single time point (p = 1.0 and 0.7, respectively). Blots are representative of n = 4 mice.

Because the Per2 protein in the Per2:Luc reporter mice is expressed as a ‘knock-in’ Per2-Luciferase fusion protein [33], we sought to validate that Per2 protein functioning was the same in the Per2::Luc reporter mice as in WT BL6 mice. To address this question, we used antibody to the Per2 protein and immunoprecipitation (IP) followed by Western blotting for two key validated circadian protein binding partners for Per2: Cry2 and Bmal1 in both WT BL6 and Per2::Luc mice (n = 3–4 each) [2]. Colon tissue samples were collected and stored in appropriate storage conditions prior to analysis. These samples were not dependent on the timeframe used for optimal colonic organoid growth (3 weeks) in analysis. **Fig 1** shows Western blotting data from these IP experiments in which no difference in Per2 protein binding to Bmal1 or Cry2 proteins was detected in the Per2::Luc mice colon tissue vs. WT BL6 colon tissue (p = 1.0 and 0.7, respectively). These data support that the Per2::Luc fusion protein exhibits similar circadian-related binding function to the WT BL6 Per2 protein.

### Circadian period, amplitude, and phase

**Fig 2:**
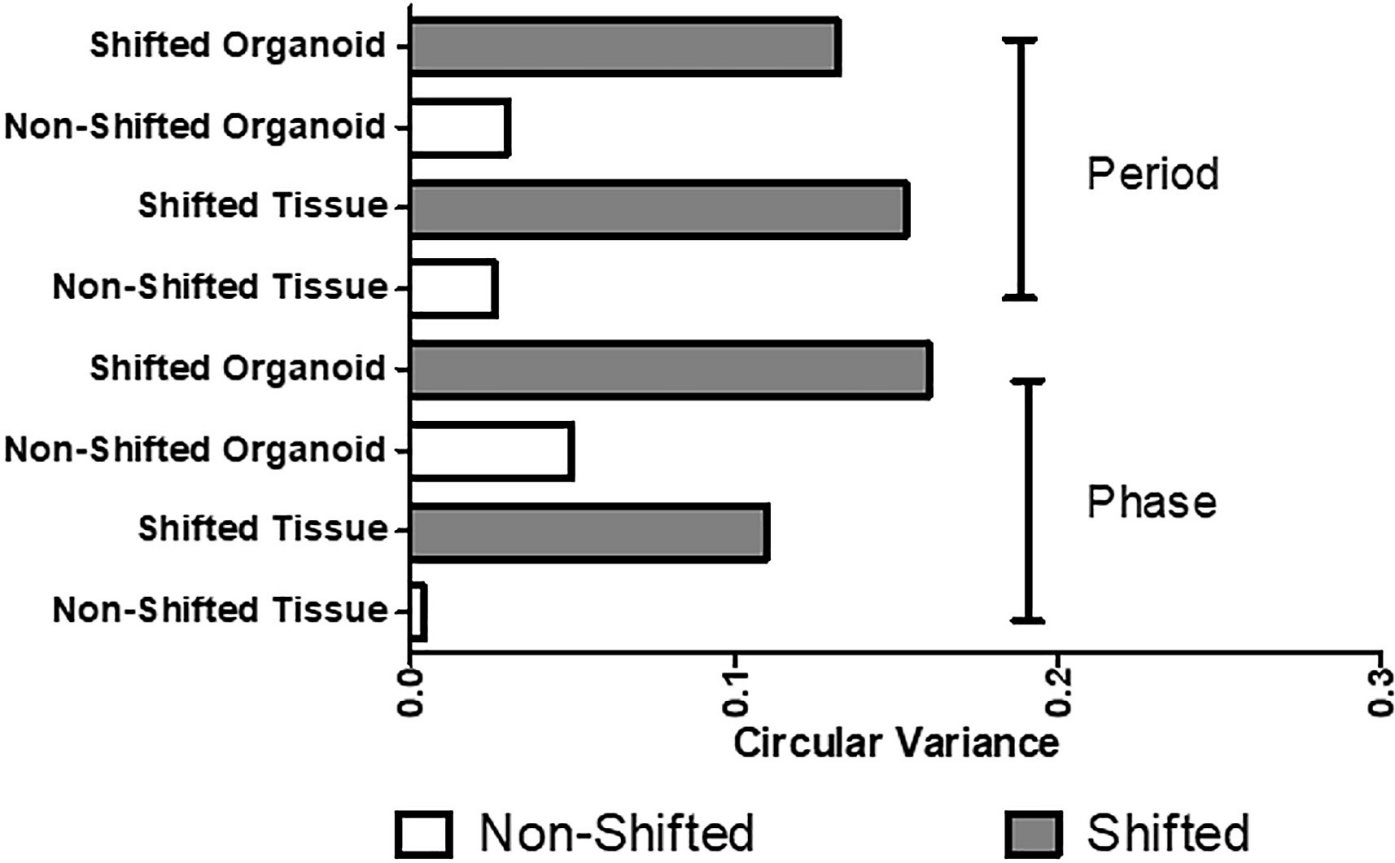
Circular variance of circadian period and phase measures is increased in LD shifted vs. non-shifted in Per2::Luc mice colon tissue and organoids. Circular variance quantifies the variability in circadian data. The top section depicts the circadian period of the colon tissue and organoids from shifted and non-shifted mice. The period of the shifted mice for both the organoids and tissue are much more variable than the periods for the non-shifted organoids and tissue. Similarly, the acrophase (phase) for the colon organoids and tissue from shifted mice has much more variability than the colon organoids and tissue from the non-shifted mice. This demonstrates that LD shifting mice creates more variability (disruption) in their colon tissue circadian clock and that colon organoids maintain the circadian rhythm disruption of the disrupted host colon tissue. Data are for n = 8-10/group.

A key aim of this study was to assess the disruption in circadian period and acrophase in mice colonic epithelium undergoing the circadian stress of chronic LD shifting and determine if these changes were stable and therefore likely to possibly promote pathology *in vivo*. Circadian oscillations of luciferase-tagged *Per2* were measured using colon tissue or colon organoids with the Kronos Dio apparatus, and periodogram was performed by FFT-NLLS. An example of the Per2::Luc activity for organoids is shown in **S3 Fig.** NS Per2::Luc period in tissue versus organoids was consistent at 26.7 ± 0.2 vs 26.5 ± 0.5 (p = 0.67) as was shifted period at 26.0 ± 0.9 vs 26.2 ± 0.3 (p = 0.70). There was not a significant difference in NS vs shifted period or phase, but chronic shifting caused Per2::Luc mice to have more variability (shortening and lengthening) in both the period and phase of Per2::Luc rhythms in the colon, as is demonstrated by an increase in circular variance of period and phase (**Fig 2**). Increased variability, defined as circadian disruption from our chronic phase shift model, in both the period and phase of these mice support that the circadian disruption observed in colonic tissue after chronic LD shifting is maintained in colonic epithelial stem cell-derived organoids 3 weeks after isolation from the shifted mice colons (as 3 weeks is the required time period for colonic organoids to grow).

### Intestinal colonic permeability

**Fig 3:**
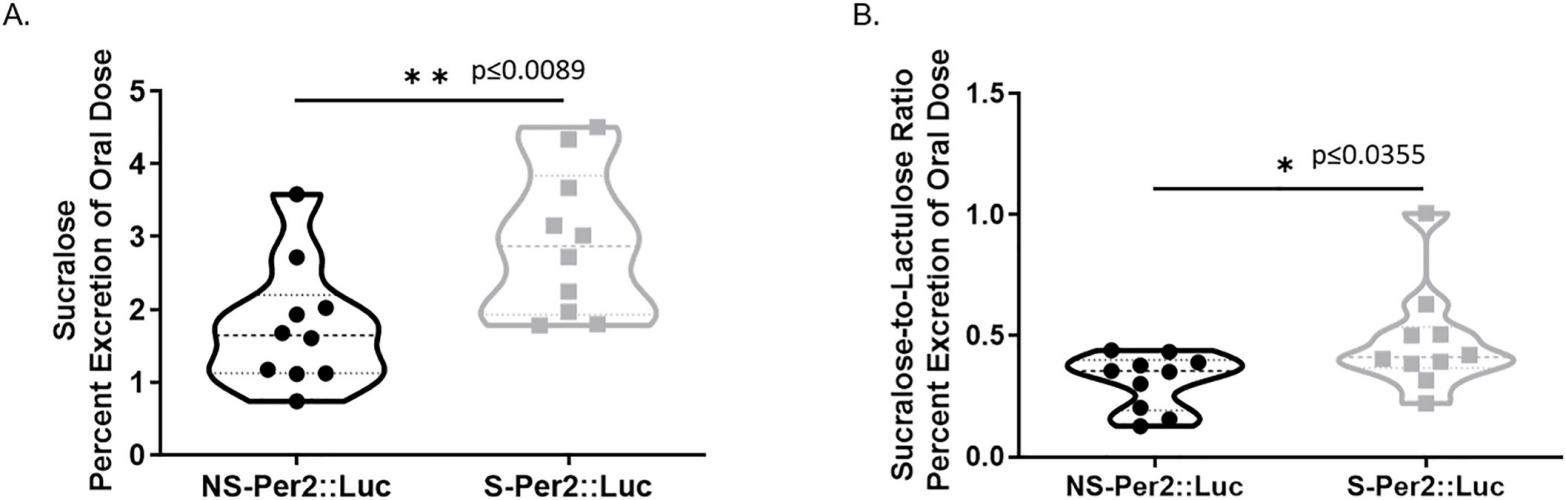
Circadian LD shifting increases colonic permeability in Per2::Luc mice. Intestinal permeability was determined by gavage of a sugar cocktail and 5h urine analysis (Methods). **(A)** The mean percent excretion of the oral dose of sucralose (permeability) for non-shifted Per2::Luc (1.769±0.271) is statistically significantly lower than that for LD shifted Per2::Luc mice (2.921±0.318, p = 0.0089). **(B)** Similarly, the mean ratio of percent excretion of sucralose to lactulose ratio (permeability) for non-shifted mice (0.3368±0.044) was significantly lower than that for the shifted mice (0.4783±0.068, p = 0.0355). *p<0.05. n = 8-10/group.

Another aim of this study was to investigate potential intestinal epithelial mechanisms through which disruption of central circadian rhythms by chronic LD shifting results in colonic leakiness as we have shown [14]. As expected from those studies, chronic LD shifting increased the intestinal permeability of both BL6 [14] and Per2::Luc mice. **Fig 3A** demonstrates that the mean percent excretion of oral dose of sucralose for shifted Per2::Luc mice (2.921±0.318) (p = 0.0130) Per2::Luc is significantly greater than for non-shifted Per2::Luc mice (1.769±0.271). This is a measure of increased colonic permeability when combined with our data that the lactulose/mannitol ratio was not significantly different [40].

**Fig 3B** shows that the excreted sucralose/lactulose ratio is also greater in shifted vs. non-shifted Per2::Luc mice. Although a ratio of sucralose to lactulose is prone to outliers, this ratio is relevant to gastrointestinal permeability due to the kinetics of the sugar probes. We have previously shown this is a more reliable measure of colonic leakiness than sucralose excretion alone [40]. Together these data strongly support that central circadian disruption by chronic LD shifting increased colonic leakiness in our shifted Per2::Luc mice as shown previously in WT BL6 mice [14].

### LD shifting alters apical junctional complex protein regulators of gut permeability in colon tissue and organoids

**Fig 4:**
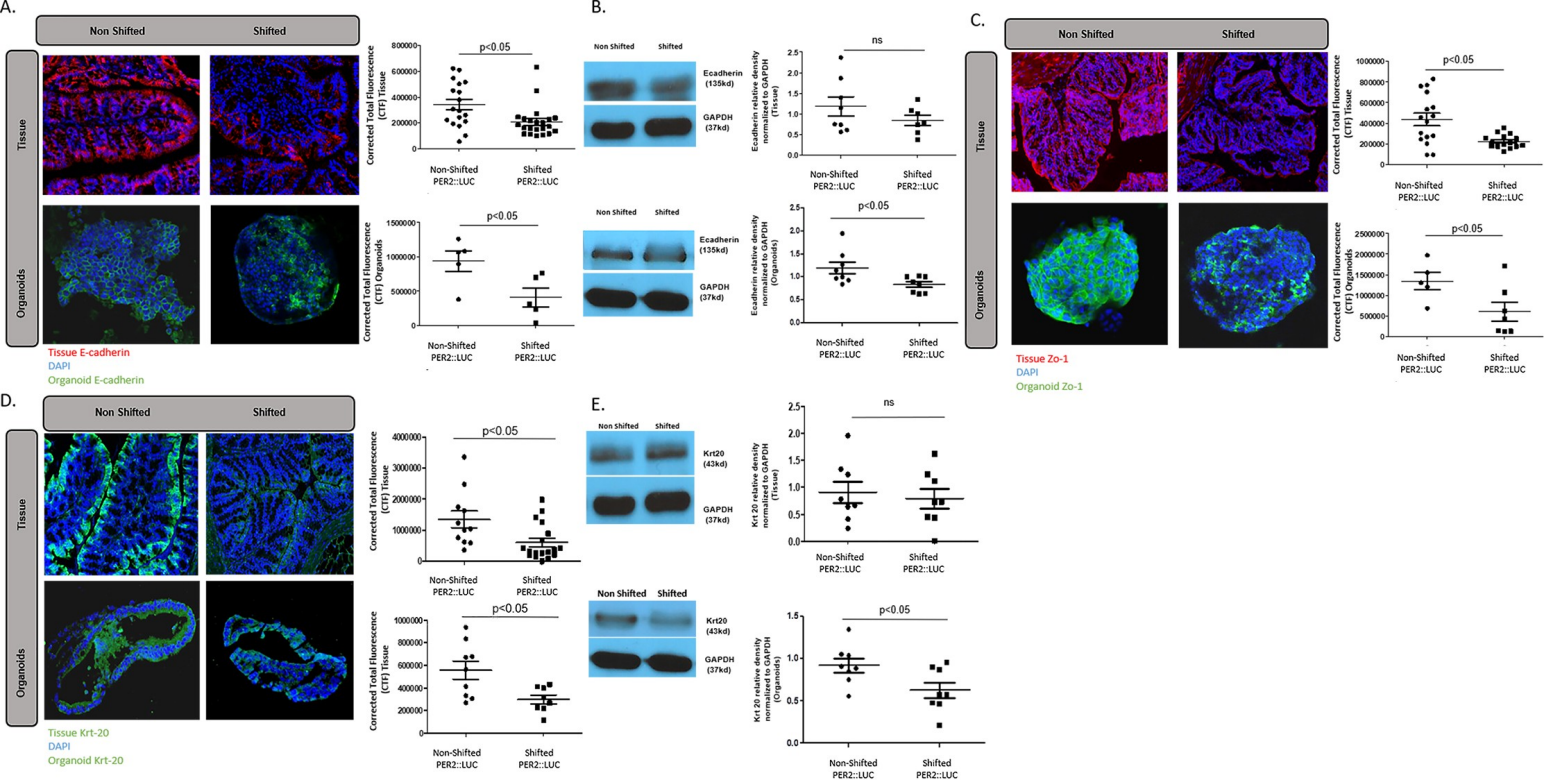
Colon tissue and organoid AJC protein expression is decreased with LD shifting in Per2::Luc mice. **(A)** Immunofluorescent staining of E-cadherin (red) in the colon tissue and organoids of shifted and non-shifted Per2::Luc mice. Both demonstrated a significant decrease in E-cadherin in the shifted mice (p<0.05). **(B)** Western blot protein quantification demonstrated a similar trend in E cadherin levels. While the difference was non-significant in the tissue, the protein levels of E-cadherin were significantly lower in the colon organoids of the shifted Per2::Luc mice. **(C)** Immunofluorescent staining of ZO-1 (green) in the colon tissue and organoids of the shifted and non-shifted Per2::LUC mice. In both organoids and tissue, the shifted mice again demonstrated a lower amount of tight junction ZO-1 in the colons of the shifted mice (p<0.05). **(D)** Immunofluorescent staining of cytoskeleton protein Krt-20 (green) in the colon tissue and organoids of shifted and non-shifted Per2::Luc mice. Both tissue and the organoids demonstrated a significant decrease in Krt-20 after shifting (p = 0.0086, 0.020). **(E)** Western blot protein quantification of Krt-20 levels in colon tissue and organoids of shifted and non-shifted Per2::Luc mice. No difference was seen between the non-shifted and shifted colon tissue levels of Krt-20, however, a lower amount was seen in the colon organoids of the shifted mice compared to those of the non-shifted mice (p<0.05). This could be due to the same dilution effect seen in section B.

Our data above (**Fig 2**) and in previous studies support that chronic LD shifting for 22 weeks results in colonic hyperpermeability in Per2::Luc mice [14]. The apical junctional complex (AJC) of intestinal epithelial cell proteins contains the tight junction protein Zonula occludens protein 1 (ZO-1) and the adherens junction protein E-cadherin that are key regulators of gut leakiness. We therefore sought to determine whether chronic 22-week LD shifting resulted in changes in expression of these two key AJC proteins that regulate permeability in colon tissue and organoids from S vs. NS mice. Our protein staining data shows both proteins were found to be clearly lower in the colon epithelial tissue and organoids from S Per2::Luc mice. **Fig 4A** shows a pattern with the E-cadherin AJP protein, with IF staining demonstrating lower amounts of E-cadherin protein in the colon tissues and colon organoids from shifted Per2::Luc mice but not in NS Per2:Luc mice colon tissue or organoids (p < .05 for each). **Fig 4B** shows Western blot data for E-cadherin with both shifted organoid and tissue samples staining show a trend towards lower E-cad protein expression, though only the shifted mice organoids demonstrate significance (p<0.05). This is possibly because organoids only include the colonic epithelial layer, while tissue samples include extraneous non-epithelial intestine that may dilute the effect of shifting on AJC proteins in tissue samples. **Fig 4C** demonstrates the IF staining of ZO-1 in colon epithelial tissue and organoids. Importantly, this figure demonstrates that the Per2:Luc mice colonic tissue exhibits a decreased ZO-1 protein phenotype that is maintained in the organoids created from shifted Per2:Luc mice colons compared to NS Per2:Luc mice colon tissue and organoids. Western blot data for ZO-1 protein was too variable possibly due to the high molecular weight of ZO-1. Finally, **Fig 4D** shows IF staining for the colon enterocyte cytoskeletal protein cytokeratin-20 (Krt-20). Changes in Krt-20 are also associated with increased colon permeability. IF staining for Krt-20 was significantly decreased in both colon tissue and organoids from shifted Per2::Luc mice (p < .05). However, once again the Western blot data for Krt-20 was only significantly lower in colon organoids (p < .05) but not colon tissue from Per2::Luc shifted mice (**Fig 4E**). The lower amounts of these three key AJC-related proteins in the colons of S Per2::Luc mice are consistent with the increased gut leakiness seen in the shifted Per2::Luc mice shown in **Fig 1**.

### Effects of chronic shiftwork on colonic epithelial protein markers in human shift workers

**Fig 5:**
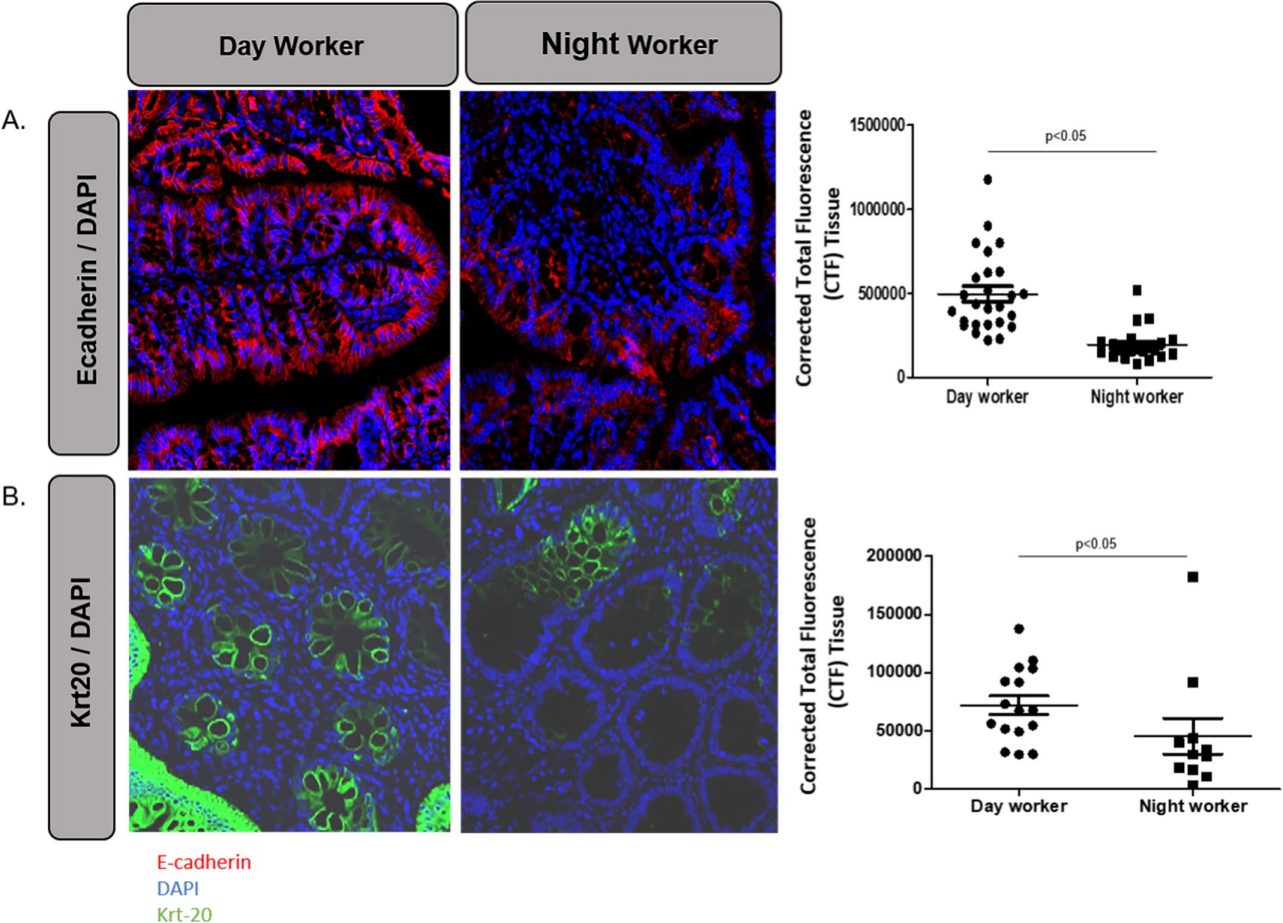
Colon tissue of a night shift worker exhibits loss of AJC permeability protein E-cadherin and Krt-20 proteins vs. a day worker’s colon tissue. Colonic biopsy tissues from a Night shift (shifted) worker and a day worker nurse were assessed for AJC protein E-cadherin and cytoskeletal protein Cytokeratin 20 (Krt-20). with IF microscopy as described in Methods. **(A)** E-cadherin levels were lower in the night shift worker compared to the day worker (p = 0.001). **(B)** Krt-20 levels were lower in the night shift worker compared to the day worker as well (p = 0.011). Data points are random field values for each sample.

Finally, in **Fig 5**, we wished to see if there was evidence for similar changes in colonic barrier proteins in humans with chronic circadian disruption due to night shift work as we found in the shifted Per2::Luc mice. We have previously shown these nurse night shift workers are prone to increased gut leakiness in response to alcohol [13]. To do this we performed a Rush IRB-approved study (see Methods) in which we obtained colonic biopsies from Rush day and night shift nurses and performed IF staining analysis for the AJC-related proteins E-cadherin and Krt-20. As seen in **Fig 5**, both proteins were significantly decreased in colon biopsy tissue from the night shift workers compared to day workers (p < .05). While these are only two human subjects, these data parallel our rodent data that colonic barrier proteins are decreased with chronic phase shifting leading to decreased colonic barrier resiliency. While cohort sizes for this experiment will need to be increased for future studies to allow for a more robust comparison of variance and shift work effects, these data serve as a “proof of concept” for our animal data for relevance in human disease.

Our **Supplemental Data** depict **S4–S7 Figs** in which we show data for staining Per2::Luc mice colon tissue and organoids for cell fate markers: Carbonic Anhydrase 2 (CA2), an enterocyte maker; Chromogranin A (ChgA) marker for enteroendocrine (EE) cells; Regenerating Islet-derived Family Member 4 (**Reg4**) a Paneth-like cell marker; and Alcian blue mucin staining, a marker for Goblet cells. Only Reg4 was significantly changed in shifted mice with an increase in shifted mice colon organoid Reg4 protein staining and Western blot (p < .05) but not significant in colon tissue. **S8 Fig** shows staining of colon tissue from the two nurse shift workers shown in **Fig 5** was not different for ChgA, Reg4 or Alcian blue cell fate markers.

## Discussion

The primary goal of this study was to investigate whether colon intestinal epithelial tissue and colonic epithelial stem cell-derived organoids maintain the host colon tissue phenotype for circadian rhythms and permeability protein-related measures. We utilized a transgenic ‘Per2::Luc gene reporter’ mouse expressing a Per2-luciferase fusion protein [33] to measure both the colon tissue and colon epithelial stem cell-derived organoid circadian clock oscillations. We found that colon organoids had similar circadian measures as colon tissue in both the non-shifted and shifted Per2::Luc mice. Both colon tissue and organoids demonstrated significant increases in variability (circadian variability is characterized as circadian disruption from our chronic 22-week light/dark shifting model) in both period and acrophase circadian measures in shifted mice. Chronic LD phase shifting also resulted in increased intestinal permeability, with both colon tissue and organoids exhibiting decreased apical junctional complex (AJC) proteins ZO-1 and E-cadherin as well as Krt-20. These markers of intestinal permeability (e.g., apical junction proteins, claudin, occludin, etc.) are under circadian regulation [49]. This supports our previous study which found that our chronic phase shift protocol disturbs circadian regulation of apical junction proteins [14].

PER2::LUC mice have been a useful model for characterization of the circadian clock. This is particularly of interest in organs like the gastrointestinal tract where frequent sampling over a 24-hour time period can be challenging. This mouse model has been used in the past to study organoid circadian phenotype in the small intestine [36]. In 2014, Moore et al. demonstrated that organoids created from the jejunum of Per2::Luc mice displayed robust circadian rhythms [36]. They found that the Per2::Luc jejunal organoids autonomously synchronized within 12 hours, without requiring a serum shock to synchronize. Interestingly, while the field has focused mainly on small intestinal enteroids, very few studies have examined colon-derived colonoids [36] and no prior studies have examined the impact of environmental circadian disruption on colonoids.

In this study, we were able to demonstrate the resilience of colon organoids in maintaining host phenotype for circadian phenotype and they also autonomously synchronized after about 24h. We also show that barrier related AJC protein phenotype immunohistology staining and protein expression from the colon tissue of shifted mice was maintained in colon stem cell derived organoids from those mice even after three weeks in organoid culture.

There are multiple lines of evidence in cell lines, animal models, and human studies that support the scientific premise that disruption of circadian rhythms in the host negatively impacts intestinal barrier integrity: (1) alcohol (0.2%) increases the expression of the circadian clock genes *Clock* and *Per2* in a human IEC line (Caco-2 cells), and knock down of *Clock* or *Per2* gene expression by siRNA prevents alcohol-induced barrier dysfunction [12], (2) chronic disruption of the central clock by either disruption of light:dark cycles or a mutation in the core circadian molecular clock (i.e., *Clock* mutant mice) increases intestinal permeability and endotoxemia [14], (3) healthy night workers who consume alcohol (0.5g/kg daily for 7 days) have increased colonic permeability compared to healthy day workers consuming alcohol, and colonic permeability inversely correlates with decreased plasma melatonin over 24h [13] (4) our lab [50] and others [51, 52] show that circadian misalignment in the host causes microbial alterations with a decrease in bacteria (*Firmicutes*) that produce short chain fatty acids like butyrate [53], and (5) the core clock machinery (*Bmal1*) regulates IEC cell regeneration and barrier homeostasis [54, 55]. It is still not clear how the different core clock genes like *Clock* or *Bmal1* impact colonic barrier homeostasis. The *Clock* mutant and *Bmal1*KO were both associated with decreased barrier function, but a *Per2* mutant with overexpression was associated with tightening of the intestinal barrier. Future studies will need to focus on how loss or gain of function of the core circadian clock can differentiate impact colonic barrier homeostasis. In addition, duration of environmental change needed to impact the circadian clock. Prior work in humans before and after long distance travel with an 8 to 10 h time shift has shown a significant alteration in intestinal microbiota with an increase in Firmicutes/Bacteroidetes ratio. This change resolved two weeks after the flight [51, 52]. Other studies have confirmed these findings [51, 52] but longer-time interventions, larger samples sizes, and studies in animals and humans are still lacking. Future studies on the duration of circadian intervention will need to consider not only intestinal epithelial cell barrier but also microbiome structure and function.

The organoid model presents multiple exciting possibilities. Organoids replicate the complete stem cell differentiation hierarchy to allow the *in vitro* study of cell differentiation [7, 55]. Organoids also provide the possibility of using biopsies from live donors as the tissue source for organoids that can be transfected in culture and analyzed at the clonal level [56]. For circadian research, the ability to utilize organoids with intrinsic circadian rhythms opens the door to further *in vitro* circadian experiments exploring disease modeling, medications, and investigate mechanisms of disease and medication effect in a much more precise manner. Ultimately, utilizing colon organoids for *in vitro* circadian studies could translate into therapeutic improvements for human GI diseases [14]. The chronobiology of the GI system is not well understood [14, 57] and organoids represent a methodological innovative breakthrough to study circadian rhythms in models of specific disease pathologies and exposures.

Another purpose of this study was to utilize Per2::Luc mice to explore the relationship between the components of the circadian rhythm and intestinal barrier function. It has previously been established that disruption of circadian homeostasis worsens colitis [58] and increases intestinal permeability in a rodent model [58]. We discovered that shifted mice have a significantly more variable (disrupted) period and acrophase in addition to a weakened intestinal barrier. The increased variability in period and acrophase of the colon seen after environmental shifting of animals has not been identified prior to this study. This deviation from normal may contribute to the mechanisms through which central circadian disruption leads to increased intestinal permeability.

The current study also had several important weaknesses. First, the time of food consumption, the main zeitgeber for colonic circadian rhythms, was not controlled in our environmental central phase shift model. This decision was based on a clear phenotype in our prior published work with a central circadian disruption model [58] and because we wanted to confirm that central circadian disruption could impact the colonic circadian rhythms disruption prior to altering food timing to impact peripheral colonic circadian rhythms. However, future studies are needed to explore the relationship of food timing and colonic circadian rhythms in this Per2::Luc model. Food timing may elicit peripheral circadian phase-shifting effects and act as an environmental entrainer [58]. The increase in circular variance we observe in our central phase shift model may be related in part to changes in time of food consumption which will be further explored. We had a small difference in central circadian timing in the BL6 and Per2::Luc mice which should be noted in comparisons between these two groups of rodents.

Another limitation to the human subject aspect of our study were numerous variables that may explain the difference we observed between shift work and non-shift work subjects. The gold standard would be to determine dim light melatonin onset (DLMO) with hourly plasma melatonin which is typically ~21:30 in humans and an accurate measure of the central circadian clock. Recruiting human subjects, carefully assessing them prior to phase assessment, measuring in lab conditions to control for environmental variables related to the central circadian clock (i.e., light), and increasing the sample size to control for other relevant biological factors (age, gender, race, etc.) is outside the scope of the current study which was mainly an animal experiment; however, the human data was included as a “proof of concept” for our animal data for relevance in human disease.

This study demonstrated that colon organoids are an excellent model for animal circadian studies. This introduces many new opportunities for circadian research in gastrointestinal disease processes. The Per2::Luc mice used in this study provided an excellent model that recapitulated the increased permeability found after LD shifting in BL6 mice and allowed for measures of circadian rhythms in the Kronos Dio apparatus. Thus, our study adds an important new model to the study of the role of circadian rhythms in gastrointestinal health and disease. The importance of circadian rhythms and circadian clock genes in physiology and pathologies continue to be reinforced, however, the mechanisms behind these phenomena are still unclear. We propose that the use of colon organoids in circadian research will provide a useful model to investigate the profound biological influence of circadian rhythms in human health and disease processes.

## Supporting information

S1 Fig Overall light/dark (LD) shifting study design. This schematic describes the 2 patterns of light and dark used in our study. The shifted mice alternated light and dark 12h timing (left) while the non-shifted mice (right) had regular 6am-6pm light and 6pm-6am dark for 22 weeks.
(TIF)

S2 Fig Central circadian period in BL6 and PER2::LUC mice. Shown are representative examples of central circadian periods (activity) determined using running wheels for both groups under dark:dark conditions as described in Methods. Both sets of mice exhibited typical curves for circadian rhythm. The Per2::Luc mice had a central circadian period of 24.10±0.16 which differed slightly from the BL6 mice who had a central circadian period of 23.81±0.067 (p = 0.013). n = 5 mice per group.
(TIF)

S3 Fig PER2::Luc bioluminescence in PER2::Luc organoids. Insertion of the luciferase reporter in the *Period* gene allows for easy assessment of Period gene expression across 24 hours making it an ideal system to study circadian. Bioluminescence expression levels of the Per2-luciferase protein in luciferin containing media is quantified between shifted and non-shifted Per2::Luc mice.
(TIF)

S4 Fig Colon carbonic anhydrase 2 (CA2) protein expression in Per2::Luc mice. Immunofluorescent staining and Western blot analysis (Methods) of Carbonic anhydrase 2 (CA2) protein (colon enterocyte marker) in the colon tissue (A/B) and organoids (C/D) of LD shifted and non-shifted Per2::Luc mice. For CA2 protein expression no clear differences were seen for shifted vs. non-shifted mice.
(TIF)

S5 Fig Colon chromogranin A (ChgA) protein expression in Per2::Luc mice. ChgA is a cell fate marker for colonic enteroendocrine (EE) cells. Immunofluorescent staining and Western blot analysis (Methods) of Chromogranin A (ChgA) in the colon tissue (A/B) and organoids (C/D) of LD shifted and non-shifted Per2::Luc mice. No differences were found in ChgA expression between shifted and non-shifted mice.
(TIF)

S6 Fig Colon regenerating islet-derived family member 4 (*Reg4*) protein expression in Per2::Luc mice. Reg4 is a cell fate marker for Paneth-like cells in the colon epithelium crypts. Immunofluorescent staining and Western blot analysis (Methods) of Reg4 protein in the colon tissue (A/B) and organoids (C/D) of LD shifted and non-shifted Per2::Luc mice. No differences were seen for Reg4 in colon tissue but Reg4 protein IF (p < .05) and WB (p < .05) data was significantly increased in organoids from shifted mice colons.
(TIF)

S7 Fig Colon alcian blue mucin (goblet cell) protein expression in Per2::Luc mice. Alcian blue stains mucin proteins produced by Goblet cells in the colon and so is a fate marker for Goblet cells. No differences were found in colon tissue or organoid alcian blue staining (Goblet cells) between shifted and non-shifted mice.
(TIF)

S8 Fig Colon protein expression of cell fate markers in day worker and night worker biopsies. ChgA is a marker for enteroendocrine (EE) cells; Reg4 is a marker for Paneth-like cells; Alcian blue is a marker for Goblet cells. Colon biopsy tissues from both a day worker and night worker were stained with Ab to these three cell fate protein markers as described in Methods. No differences were seen in the expression of these three cell fate proteins in the biopsies from these subjects.
(TIF)

S1 Table List of primary antibodies for immunofluorescent staining. (TIF)

S2 Table List of primary antibodies for immunoblotting. (TIF)

S1 Raw images (PDF)
