# Supplementary figures and images for: Circadian misalignment by environmental light/dark shifting causes circadian disruption in colon
Source: PLoS One. 2021 Jun 4;16(6):e0251604. doi: 10.1371/journal.pone.0251604 (PMC8177509; doi:10.1371/journal.pone.0251604)

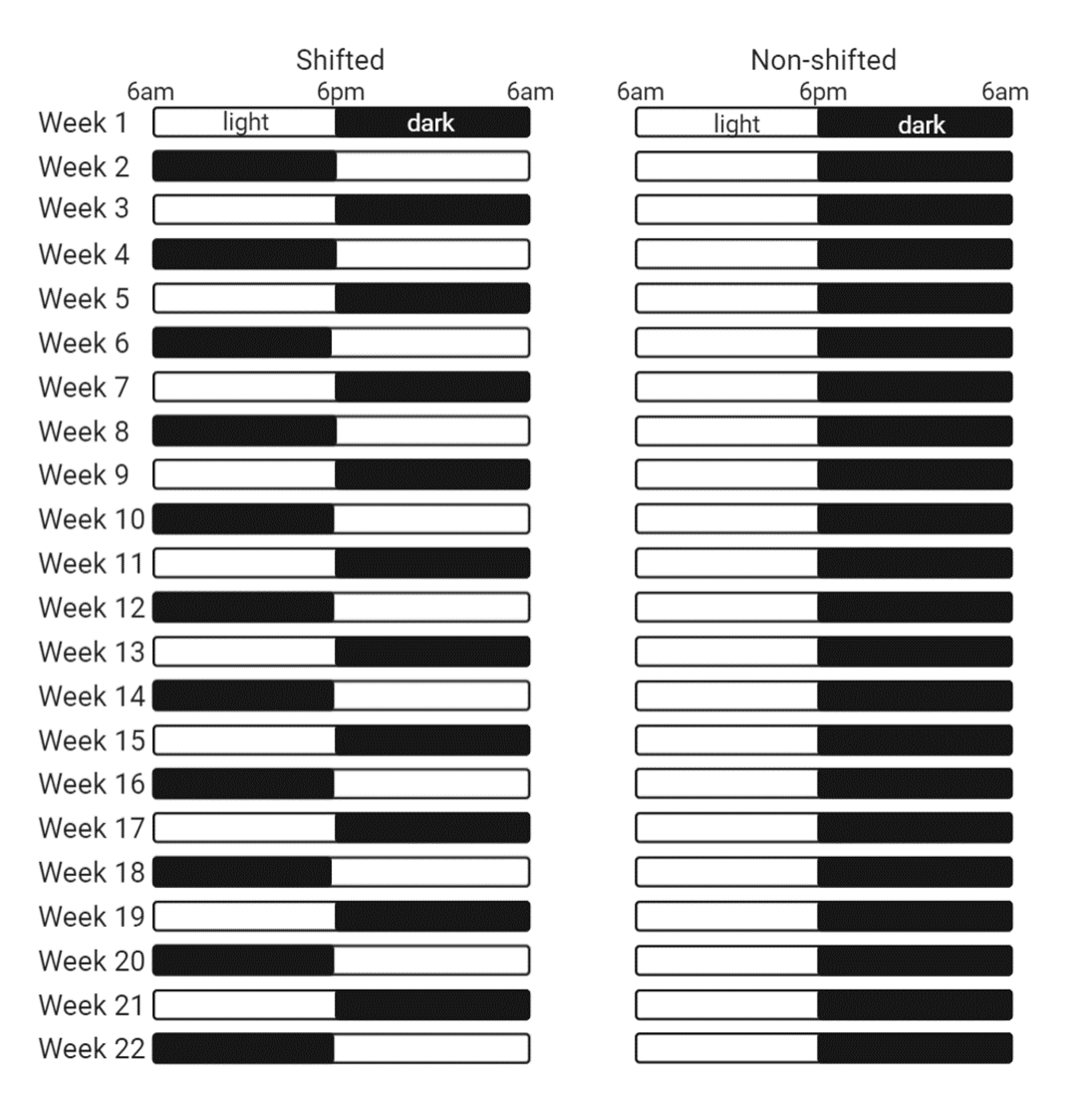

Supplement: S1 Fig — This schematic describes the 2 patterns of light and dark used in our study. The shifted mice alternated light and dark 12h timing (left) while the non-shifted mice (right) had regular 6am-6pm light and 6pm-6am dark for 22 weeks. (TIF) [file pone.0251604.s001.tif]

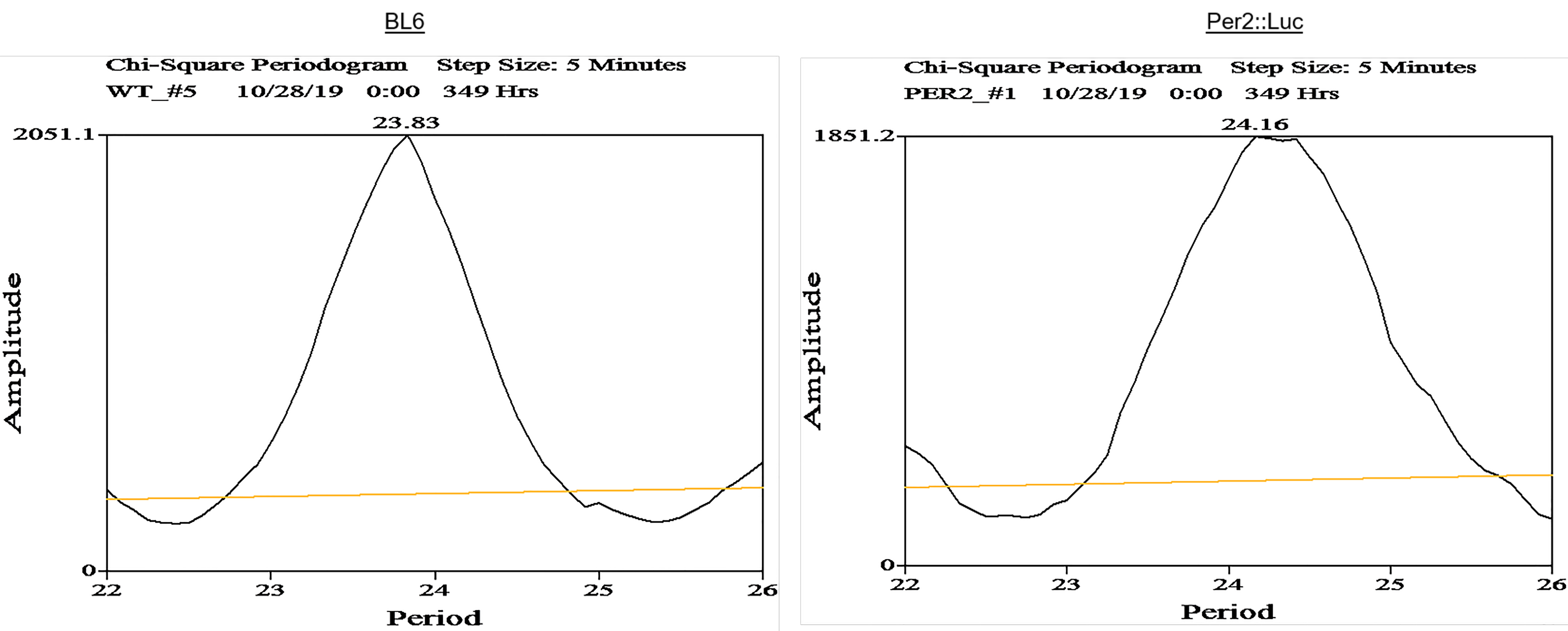

Supplement: S2 Fig — Shown are representative examples of central circadian periods (activity) determined using running wheels for both groups under dark:dark conditions as described in Methods. Both sets of mice exhibited typical curves for circadian rhythm. The Per2::Luc mice had a central circadian period of 24.10±0.16 which differed slightly from the BL6 mice who had a central circadian period of 23.81±0.067 (p = 0.013). n = 5 mice per group. (TIF) [file pone.0251604.s002.tif]

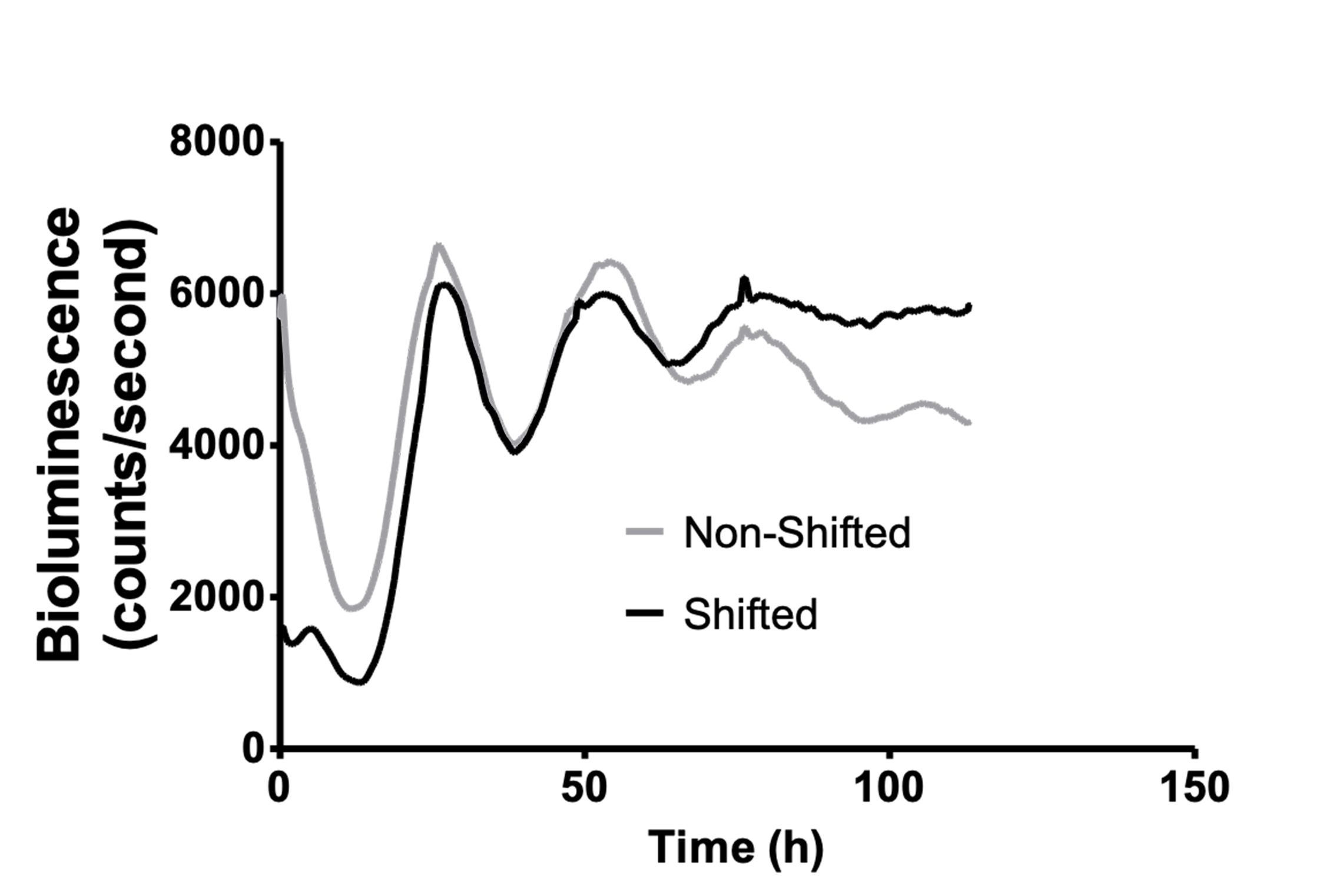

Supplement: S3 Fig — Insertion of the luciferase reporter in the Period gene allows for easy assessment of Period gene expression across 24 hours making it an ideal system to study circadian. Bioluminescence expression levels of the Per2-luciferase protein in luciferin containing media is quantified between shifted and non-shifted Per2::Luc mice. (TIF) [file pone.0251604.s003.tif]

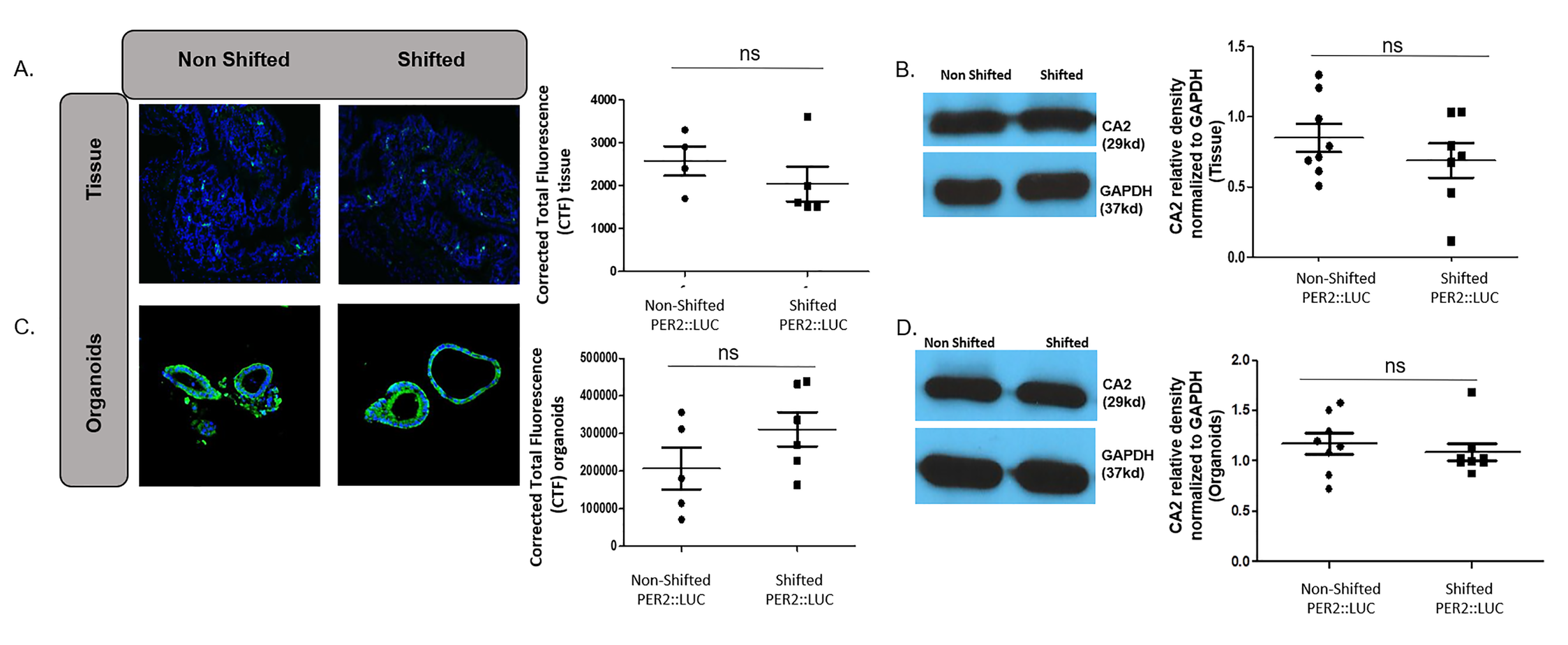

Supplement: S4 Fig — Immunofluorescent staining and Western blot analysis (Methods) of Carbonic anhydrase 2 (CA2) protein (colon enterocyte marker) in the colon tissue (A/B) and organoids (C/D) of LD shifted and non-shifted Per2::Luc mice. For CA2 protein expression no clear differences were seen for shifted vs. non-shifted mice. (TIF) [file pone.0251604.s004.tif]

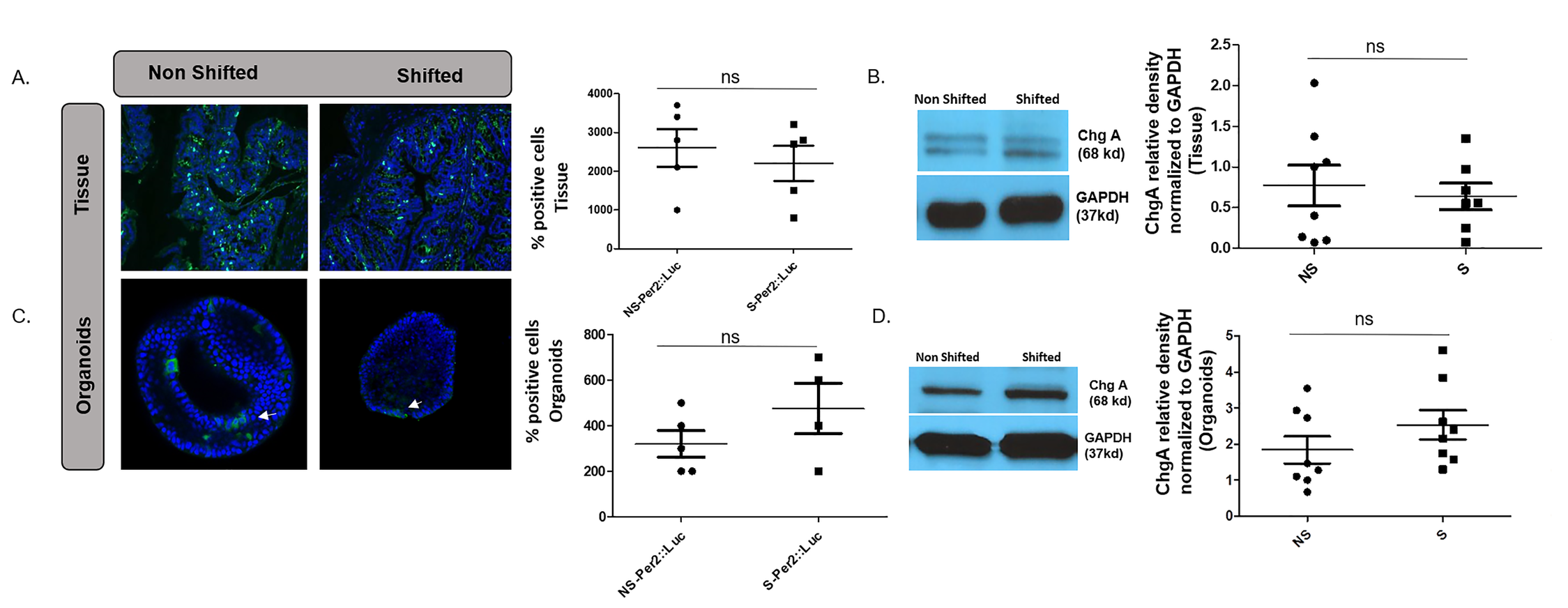

Supplement: S5 Fig — ChgA is a cell fate marker for colonic enteroendocrine (EE) cells. Immunofluorescent staining and Western blot analysis (Methods) of Chromogranin A (ChgA) in the colon tissue (A/B) and organoids (C/D) of LD shifted and non-shifted Per2::Luc mice. No differences were found in ChgA expression between shifted and non-shifted mice. (TIF) [file pone.0251604.s005.tif]

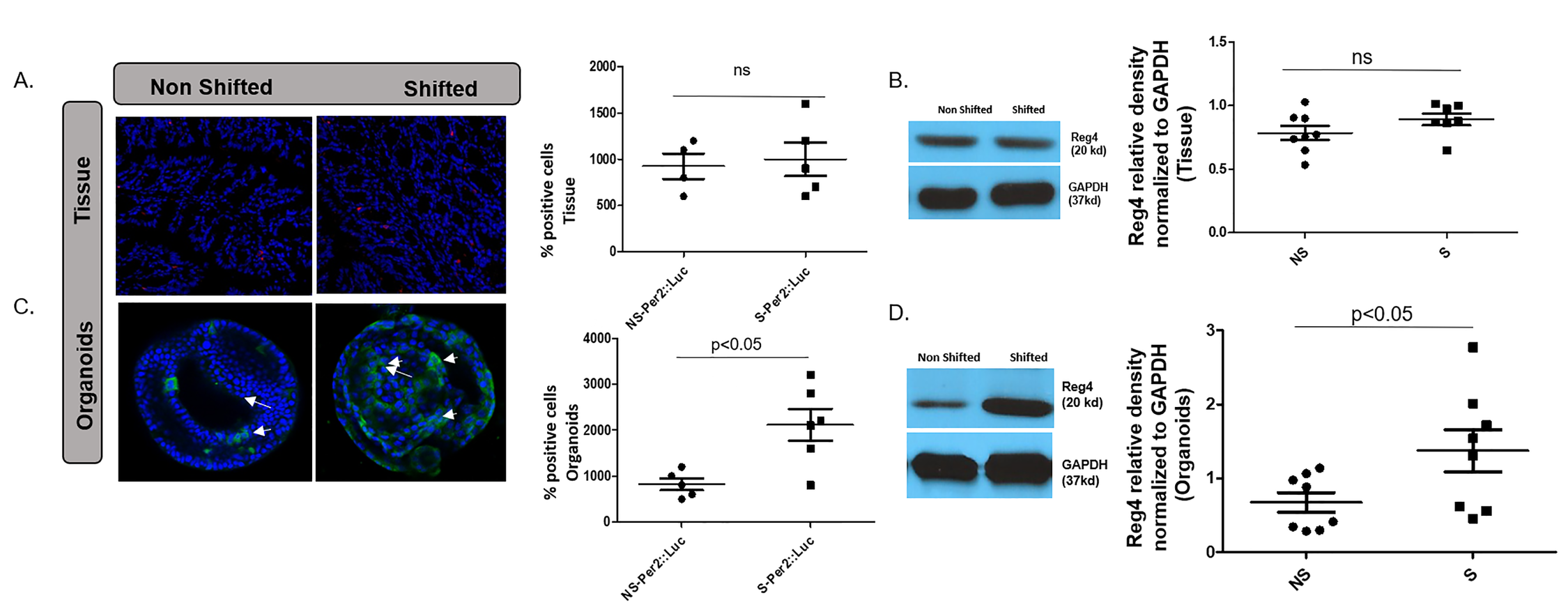

Supplement: S6 Fig — Reg4 is a cell fate marker for Paneth-like cells in the colon epithelium crypts. Immunofluorescent staining and Western blot analysis (Methods) of Reg4 protein in the colon tissue (A/B) and organoids (C/D) of LD shifted and non-shifted Per2::Luc mice. No differences were seen for Reg4 in colon tissue but Reg4 protein IF (p < .05) and WB (p < .05) data was significantly increased in organoids from shifted mice colons. (TIF) [file pone.0251604.s006.tif]

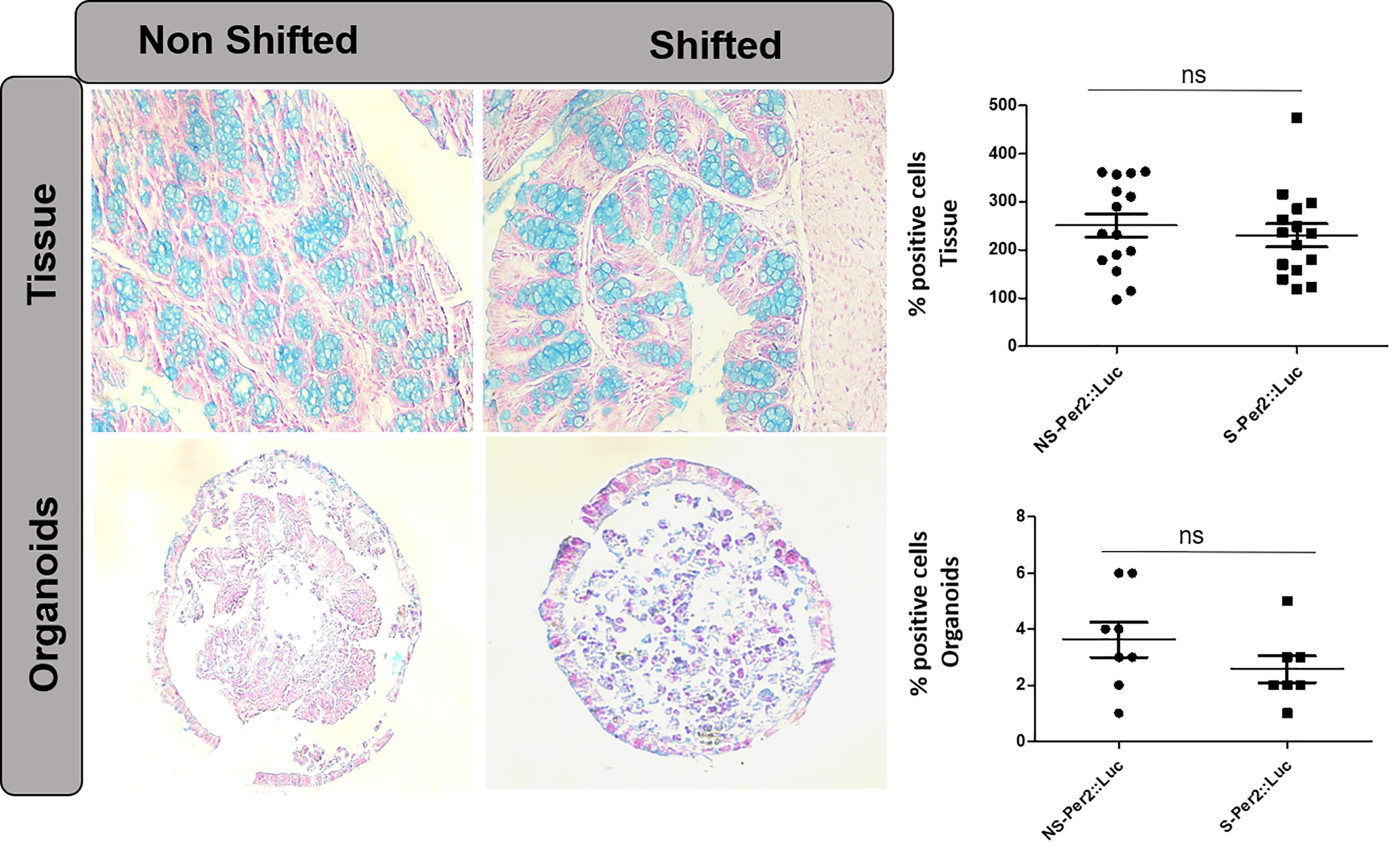

Supplement: S7 Fig — Alcian blue stains mucin proteins produced by Goblet cells in the colon and so is a fate marker for Goblet cells. No differences were found in colon tissue or organoid alcian blue staining (Goblet cells) between shifted and non-shifted mice. (TIF) [file pone.0251604.s007.tif]

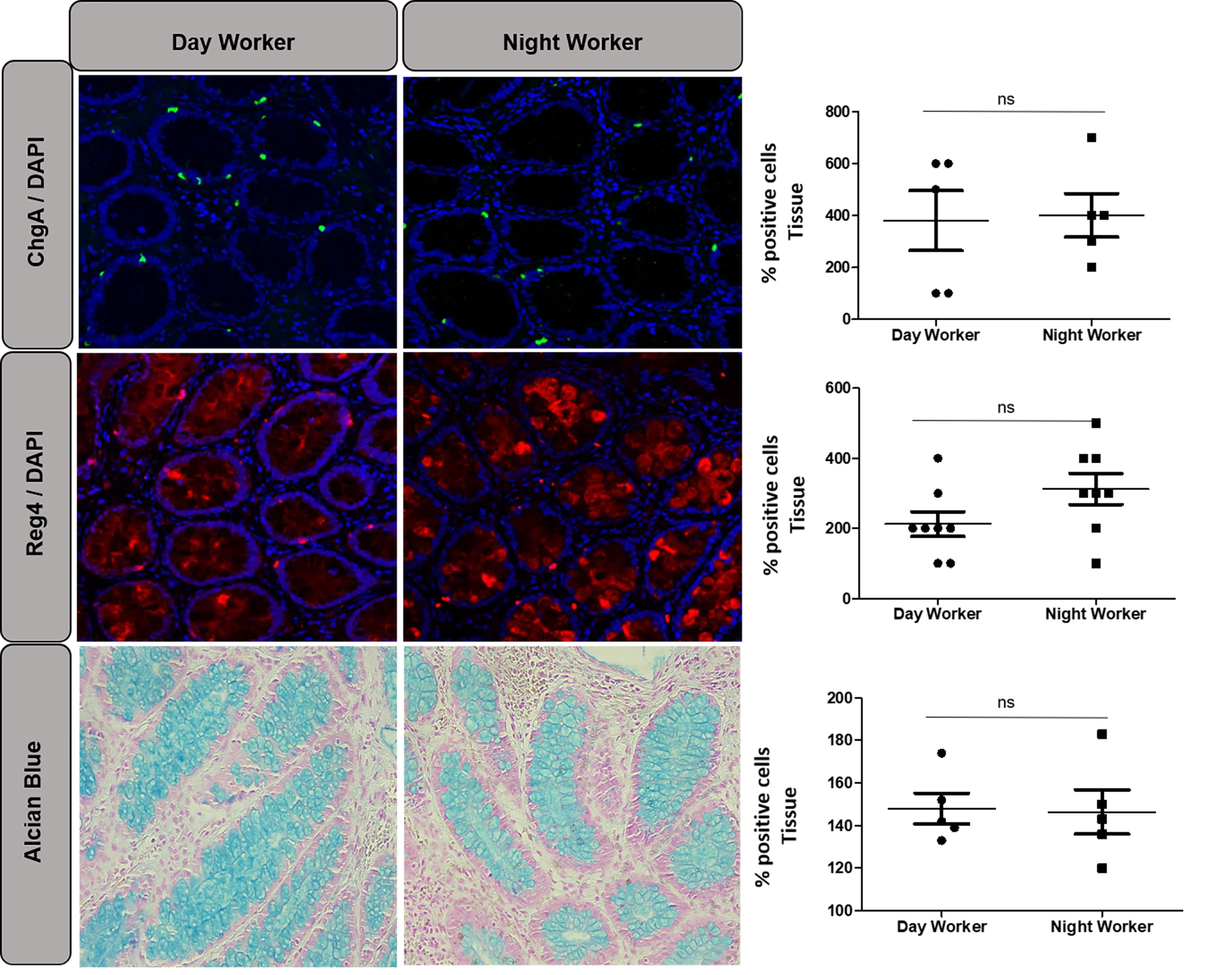

Supplement: S8 Fig — ChgA is a marker for enteroendocrine (EE) cells; Reg4 is a marker for Paneth-like cells; Alcian blue is a marker for Goblet cells. Colon biopsy tissues from both a day worker and night worker were stained with Ab to these three cell fate protein markers as described in Methods. No differences were seen in the expression of these three cell fate proteins in the biopsies from these subjects. (TIF) [file pone.0251604.s008.tif]

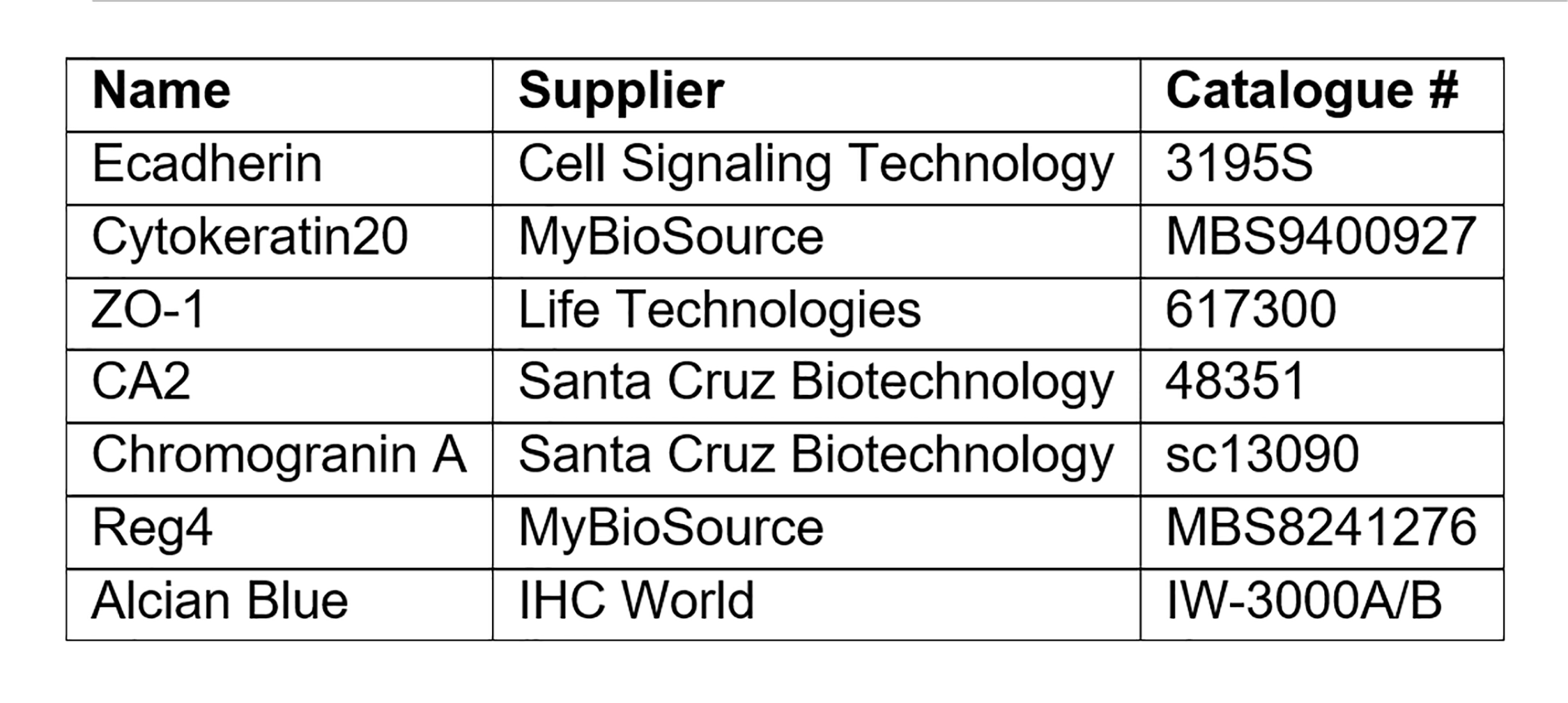

Supplement: S1 Table — (TIF) [file pone.0251604.s009.tif]

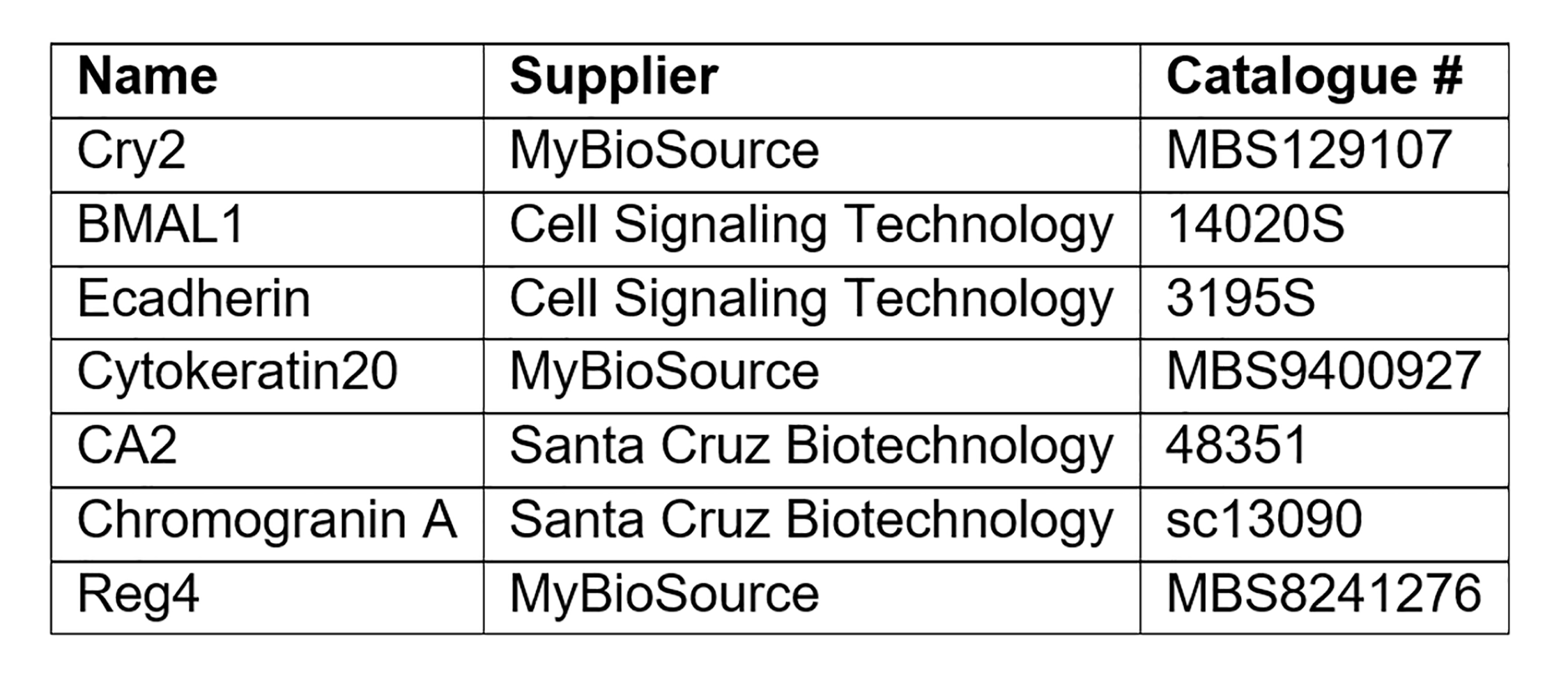

Supplement: S2 Table — (TIF) [file pone.0251604.s010.tif]
