# Supplementary material for: Circadian misalignment by environmental light/dark shifting causes circadian disruption in colon
Source: PLoS One. 2021 Jun 4;16(6):e0251604. doi: 10.1371/journal.pone.0251604 (PMC8177509; doi:10.1371/journal.pone.0251604)
Supplement: S1 Raw images — (PDF) [file pone.0251604.s011.pdf]

Fig 1. Colon tissue Per2::Luc mice Per2 protein displays typical interaction with Cry2 and Bmal1 circadian proteins. [i]

Fig 1 (Left)

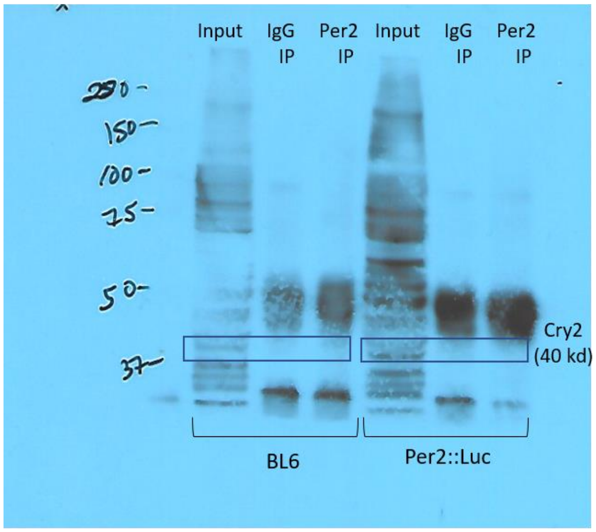

Fig 1 (Right)

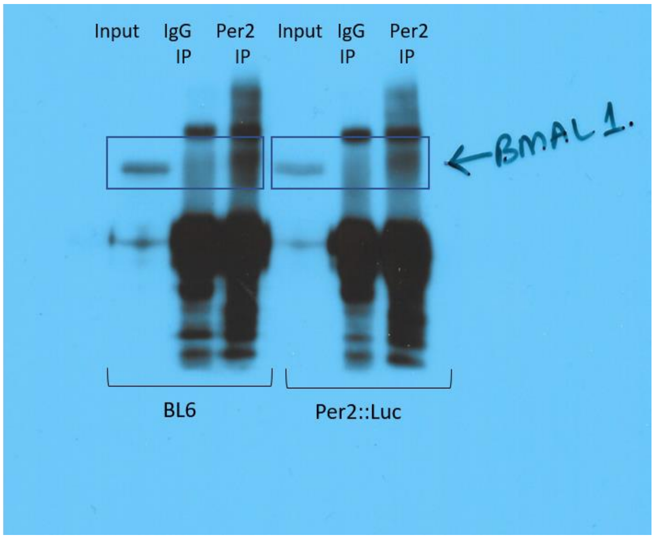

Figure 4. Colon tissue and organoid AJC protein expression is decreased with LD shifting in Per2::Luc mice.

Fig 4 B. (top)

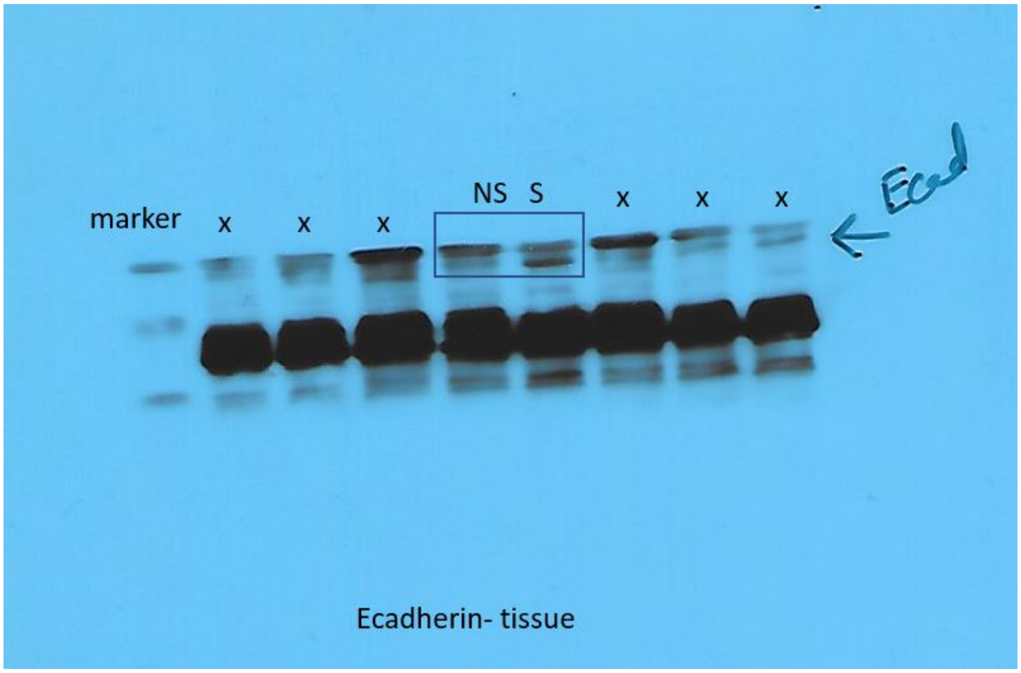

Fig 4 B and E.

(top, GAPDH)

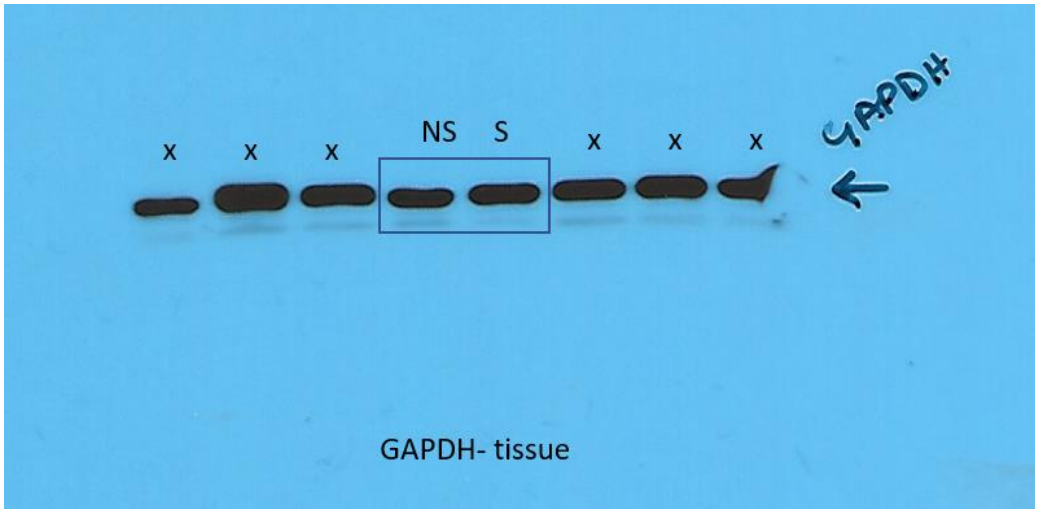

Fig 4 E. (top)

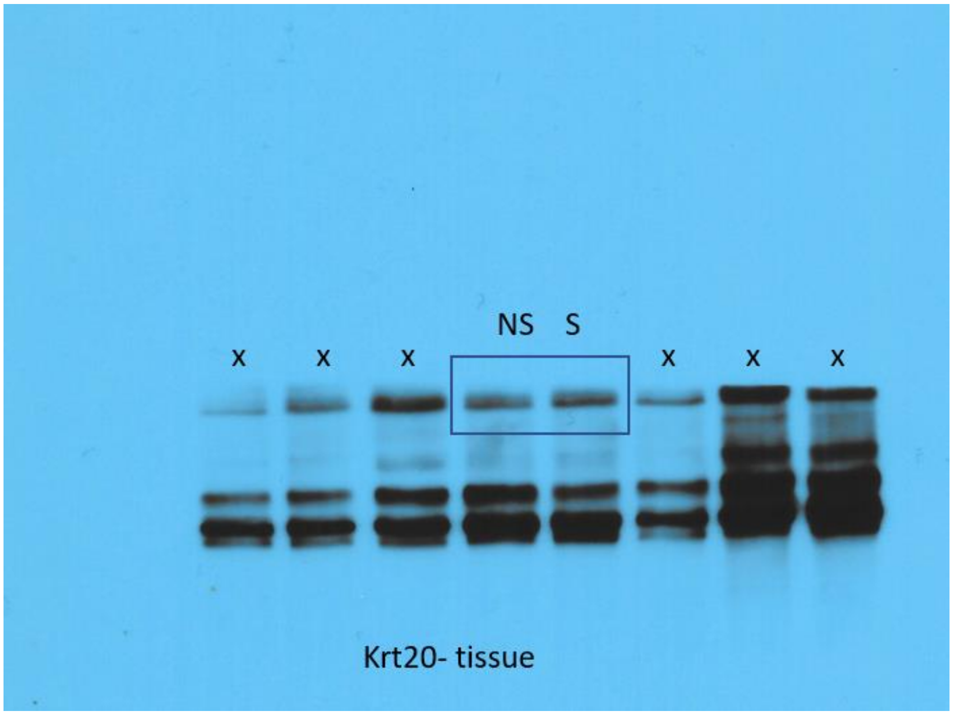

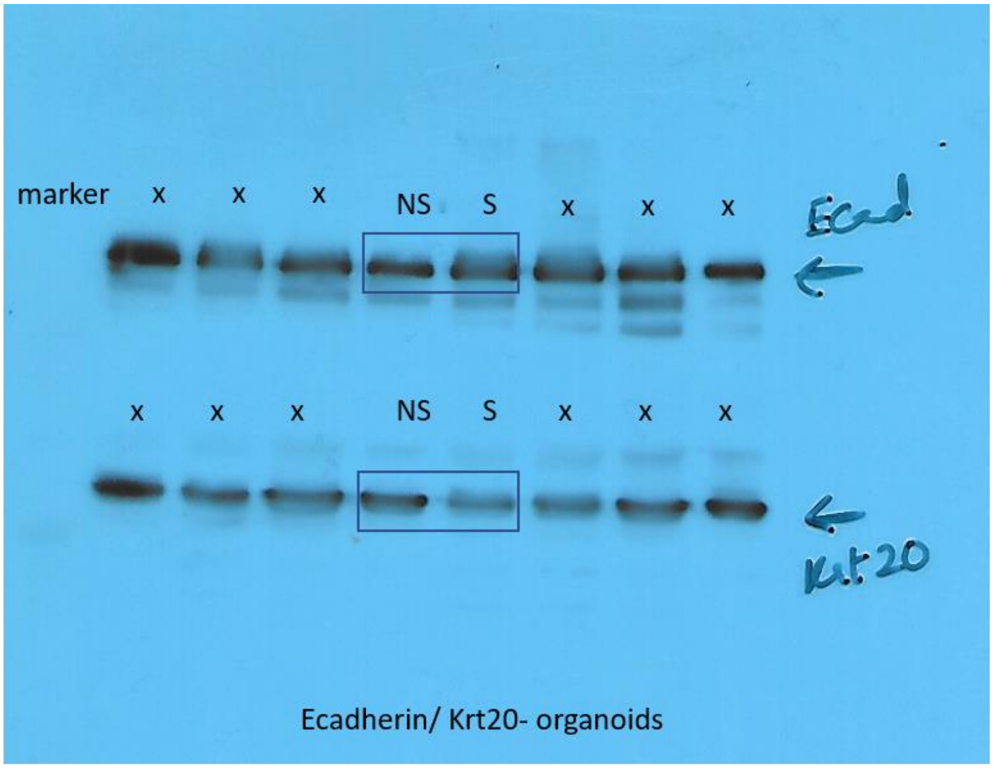

Fig 4 B.

(bottom, E cadherin)

Fig 4 E.

(bottom, Krt20)

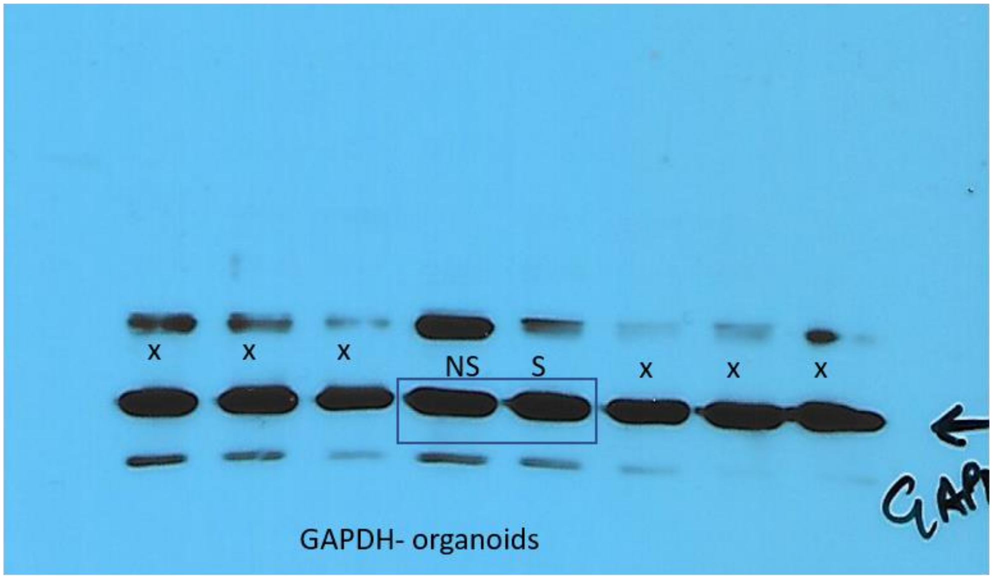

Fig 4 B and E.

(bottom, GAPDH)

S4 Fig: Colon carbonic anhydrase 2 (CA2) protein expression in Per2::Luc mice.

S4 Fig B. (top)

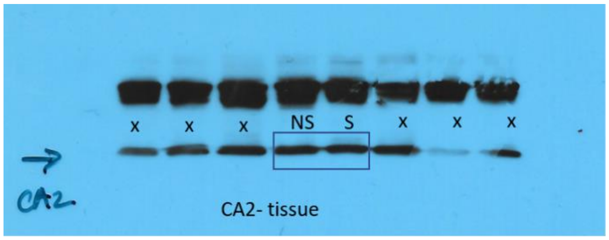

S4 Fig D. (top)

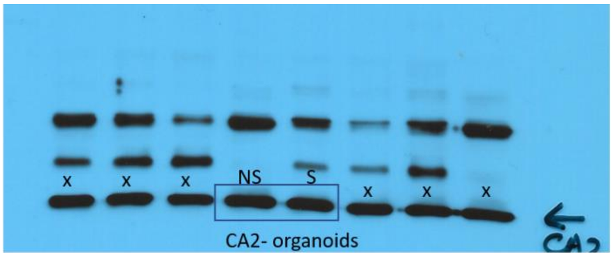

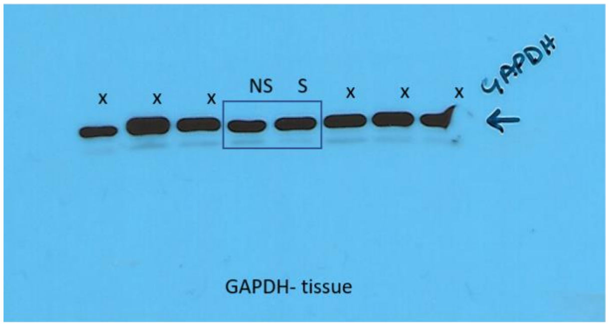

S4 Fig B. (bottom)

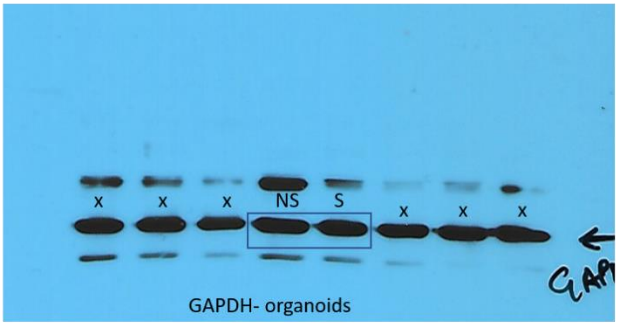

S4 Fig D. (bottom)

S5 Fig: Colon chromogranin A (ChgA) protein expression in Per::Luc mice.

S5 Fig B. (top)

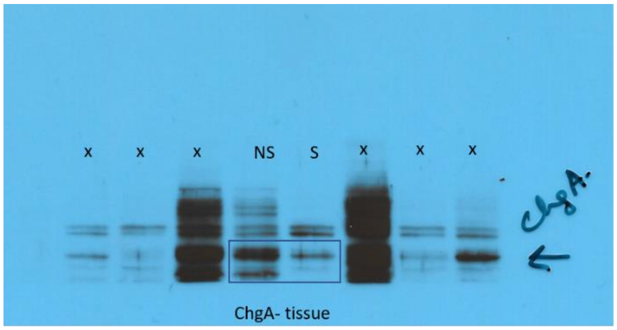

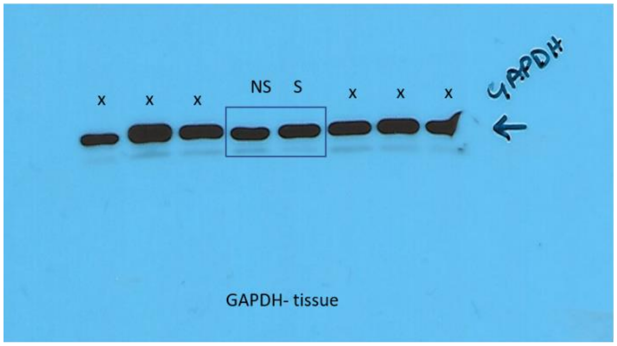

S5 Fig B. (bottom)

S5 Fig D. (top)

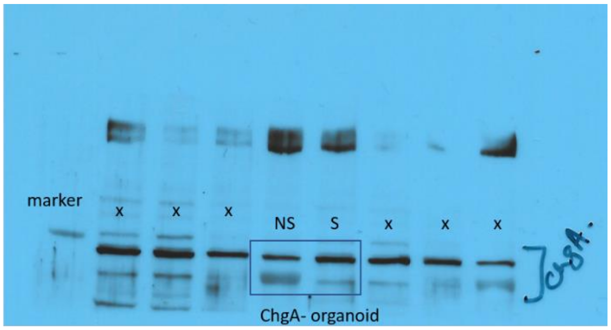

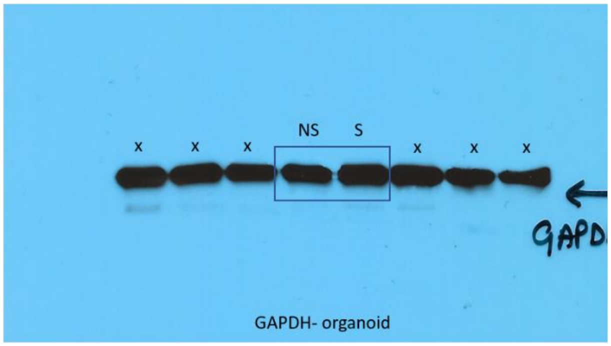

S5 Fig D. (bottom)

S4 Fig: Colon regenerating islet-derived family member 4 (*Reg4*) protein expression in Per::Luc mice.

S4 Fig B. (top)

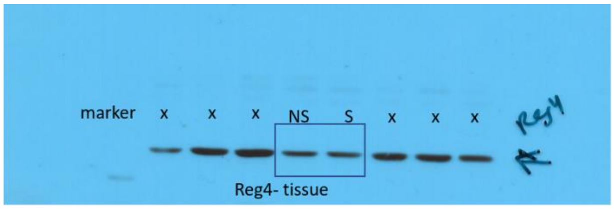

S4 Fig D. (top)

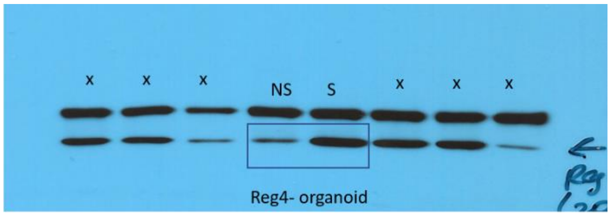

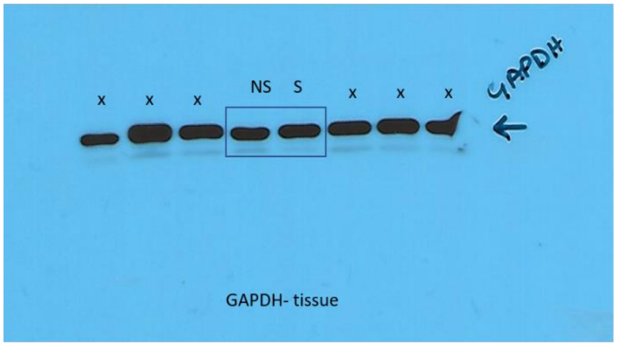

S4 Fig B. (bottom)

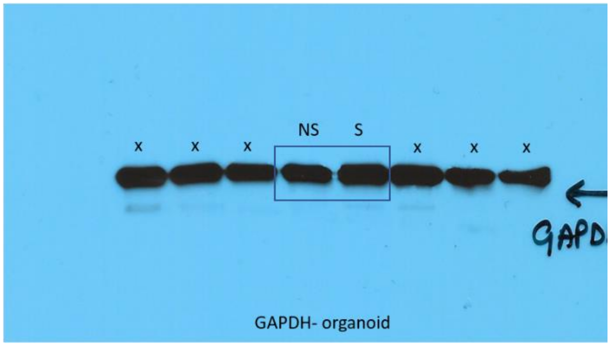

S4 Fig D. (bottom)

---

[i] For western blot analysis, protein visualization was performed on autoradiography film (HyBlot CL, Denville Scientific, Metuchen, NJ). Optical density was determined via densitometric analysis using Image J Software (NIH, Bethesda, MD).
